# The efficacy and safety of Chinese herbal medicine for mild cognitive impairment: a systematic review and meta-analysis of randomized placebo-controlled trials

**DOI:** 10.3389/fphar.2024.1341074

**Published:** 2024-02-15

**Authors:** Lingling Liu, Claire Shuiqing Zhang, Anthony Lin Zhang, Yefeng Cai, Charlie Changli Xue

**Affiliations:** ^1^ China-Australia International Research Centre for Chinese Medicine, School of Health and Biomedical Sciences, RMIT University, Melbourne, VIC, Australia; ^2^ The Second Affiliated Hospital of Guangzhou University of Chinese Medicine, Guangdong Provincial Hospital of Chinese Medicine and Guangdong Provincial Academy of Chinese Medical Sciences, Guangzhou, Guangdong, China

**Keywords:** Chinese herbal medicine, efficacy, meta-analysis, mild cognitive impairment, safety, systematic review

## Abstract

**Objective:** Effective and safe treatments for mild cognitive impairment (MCI) are limited. Chinese herbal medicine (CHM) is commonly used in China to manage MCI. However, its efficacy and safety remain uncertain. This review aims to evaluate the efficacy and safety of CHM for MCI.

**Methods:** Nine databases were searched from their inceptions to January 2023. Randomized, placebo-controlled trials of oral CHM for MCI were included. Study quality was assessed using the Cochrane risk-of-bias tool 2.0, and the certainty of evidence was evaluated via the GRADE approach.

**Results:** Thirteen studies, involving 1,043 participants, were analyzed. Most of the studies (10 out of 13) were associated with “some concerns” regarding the overall risk of bias. Meta-analyses results indicated that CHM significantly improved cognitive function compared to placebo in terms of Mini-Mental State Examination (MMSE) (MD: 1.90 [1.22, 2.58], I^2^ = 87%, 11 studies, 823 participants) and Montreal Cognitive Assessment (MoCA) (MD: 2.88 [1.69, 4.06], I^2^ = 81%, 3 studies, 241 participants). The certainty of evidence for MMSE was assessed as “moderate”, while it was “low” for MoCA. One study did not report adverse events (AEs), one study reported no statistical difference between the groups in terms of AEs, and 11 studies provided detailed numbers of AE cases where gastrointestinal symptoms were the most commonly reported AEs. Two studies reported no SAEs among participants and one study found no significant difference in SAEs proportions between groups. The meta-analysis revealed no significant difference in AEs between the two groups (RR: 1.31 [0.92, 1.87), I^2^ = 0%, 11 studies, 839 participants). The cognitive-enhancing function of commonly used herbs (*Panax ginseng* C.A.Mey., *Acorus calamus* var. *angustatus* Besser, and *Polygala tenuifolia* Willd.) may be attributed to mechanisms including antioxidant, anti-apoptotic, anti-neurotoxic, anti-cytotoxic, and anti-inflammatory actions.

**Conclusion:** Chinese herbal medicine holds potential as an effective intervention to improve cognitive function in MCI patients, supported by meta-analyses evidence of low to moderate certainty. Although current data suggests CHM is generally safe, caution is advised due to the lack of AE reporting or detailed information in some instances.

**Systematic Review Registration**: https://www.crd.york.ac.uk/prospero/display_record.php?RecordID=400292, identifier [CRD42023400292].

## 1 Introduction

Mild cognitive impairment (MCI) constitutes an intermediary phase between typical cognitive changes associated with aging and the manifestation of clinical dementia, representing a preclinical stage of cognitive decline that falls below the threshold for a formal dementia diagnosis (Petersen, 2011). This transitional state assumes particular prominence within the aging demographic, with reported prevalence figures ranging from 6.7% to 25.2% among individuals aged 60–84 years (Petersen et al., 2018). People with MCI had a higher risk of dementia conversion than the age-matched non-MCI population, and the average rate of progression from MCI to dementia has been reported to be 10%–15% each year (Roberts and Knopman, 2013; Langa and Levine, 2014). The etiology and pathogenesis of MCI emerge as intricate and multifarious, enlisting an assortment of factors spanning degenerative, vascular, metabolic, traumatic, psychiatric, medications and others (DeCarli, 2003; Winblad et al., 2004; Mufson et al., 2012; Jongsiriyanyong and Limpawattana, 2018).

Given that MCI is considered a precursor to dementia, it is regarded as a “critical window of opportunity” for early intervention, allowing the potential to delay the onset of dementia (Anderson, 2019). While certain anti-amyloid treatments exhibited efficacy in slowing the clinical progression of early Alzheimer’s disease during clinical trials, their application for MCI is restricted by stringent indications, such as age, comorbid conditions, laboratory or neuroimaging findings (Pittock et al., 2023; Sims et al., 2023; Van Dyck et al., 2023). Moreover, the high cost and the risk of serious side effects, such as amyloid-related imaging abnormalities with edema or effusions, may limit the broad utilization of this treatment at its current stage (Alzheimer’s Association, 2023; U.S. Food and Drug Administration, 2023). The available evidence on the clinical benefits and potential harms of these anti-amyloid treatments for MCI is still limited, indicating the need for more confirmatory trials and post-marketing adverse effect surveillance programs (Cummings et al., 2023; Wahlberg et al., 2023; Watt et al., 2023; Kaur et al., 2024). Researchers suggest that a treatment paradigm characterized by a multifactorial mode of action could offer a more pragmatic approach to address the heterogeneous pathologies observed in MCI (Kasper et al., 2020). Given the limitations of existing treatments for MCI, there is an urgent need to develop other effective and safe therapies to assist with current MCI management.

Chinese herbal medicine (CHM) has been used to manage cognitive impairment in China for a long history (May and Feng, 2018; May and Feng, 2020). A recent network pharmacology study indicates that, CHM exhibits considerable potential for treating MCI, which may attributed to various mechanisms including anti-inflammatory, antioxidant, anti-apoptotic, anti-amyloid-beta toxicity, cholinergic system regulation, and neuroprotective effects (Chang et al., 2022). Previous systematic reviews have acknowledged the potential of CHM in enhancing cognitive function among patients with MCI (Dong et al., 2016; Dong et al., 2019; Wang et al., 2021; Liang et al., 2022). These reviews encompassed diverse comparisons such as CHM vs. no treatment, CHM vs. Western medicine, or CHM in combination with Western medicine vs. Western medicine alone. However, all of these meta-analyses focused on effectiveness rather than efficacy. Furthermore, recent clinical studies conducted outside of China (Park et al., 2019; Chen et al., 2021; Shin et al., 2021) were not included in those reviews. Therefore, to address these research gaps, our systematic review was designed to specifically evaluate the efficacy in terms of cognitive function and the safety profile of CHM for the treatment of MCI, when compared to a placebo.

## 2 Methods

This systematic review was conducted according to the Cochrane Handbook (Higgins et al., 2022) and was reported according to the Preferred Reporting Items for Systematic Reviews and Meta-Analyses (PRISMA) guideline (Page et al., 2021). The PRISMA checklist is provided in Supplementary Table S1. The study protocol was registered at the PROSPERO international prospective register of systematic reviews (https://www.crd.york.ac.uk/prospero/display_record.php?RecordID=400292), and the registered ID is CRD42023400292. Randomized controlled trials (RCTs) comparing oral CHM with placebo were included in this review.

### 2.1 Eligibility criteria

Studies that met all of the following criteria were included in this systematic review:

Participants: Patients diagnosed with any type of MCI using standardized diagnostic criteria or based on the clinicians’ assessment. Patients with coexisting conditions, such as cerebrovascular disease, were not excluded from consideration in this review.

Interventions: Any orally administered CHM. Studies evaluated single compounds extracted from certain herbs, such as the standardized extract of *Ginkgo biloba* L., were not included in this review since these herbs were not classified as traditional CHM (DeFeudis, 2003). Usual care for underlying diseases was allowed if the same treatments were applied to both the CHM and placebo groups, except for any other types of Chinese medicine therapies, anti-dementia drugs or other therapies aimed at improving cognitive function (e.g., cognitive training).

Controls: Only placebo-controlled trials were included. Co-interventions were allowed if they were the same as those used in the intervention group.

Outcomes: Studies reporting at least one of the following outcomes at the end of treatment were included: scores of Mini-Mental State Examination (MMSE) and Montreal Cognitive Assessment (MoCA). This review also analyzed adverse events (AEs) if the original RCTs reported this outcome.

Study design: Only RCTs were included.

### 2.2 Search strategy

Two reviewers (LL and CSZ) independently searched nine databases: PubMed, Excerpta Medica Database (Embase), Cochrane Central Register of Controlled Trials (CENTRAL) (including the Cochrane Library), Cumulative Index of Nursing and Allied Health Literature (CINAHL), the Allied and Complementary Medicine Database (AMED), China Biomedical Literature (CBM), China National Knowledge Infrastructure (CNKI), Wanfang and Chongqing VIP (CQVIP) databases, from their respective inceptions to January 2023. No restrictions were placed on the language of publication. The search terms were the keywords and their synonyms of MCI, CHM, and RCT. Details of the search strategy employed on searching nine databases are presented in Supplementary Text S1. In addition, references from published systematic reviews on Chinese medicine for MCI were hand-searched.

### 2.3 Study selection and data extraction

Two reviewers (LL and CSZ) independently screened the articles’ titles and abstracts against the pre-defined selection criteria, excluding irrelevant studies and duplicates. The full-text articles of potential studies were then retrieved for further screening. Any discrepancy between these two reviewers was resolved through discussion with a third reviewer (ALZ).

For data extraction, two independent reviewers (LL and CSZ) extracted information from each eligible study, including sample size, characteristics of participants, details of intervention and control, duration of treatment and follow-up, and clinical outcomes data.

### 2.4 Risk of bias assessment

The methodological quality of each study was independently evaluated by two reviewers (LL and CSZ) using the Version 2 of the Cochrane risk-of-bias tool for randomized trials (RoB 2) tool (Higgins et al., 2022). Any disagreement between these two reviewers was resolved by discussing with a third reviewer (ALZ). The RoB 2 assesses different aspects of trial design, conduct, and reporting compared with the original RoB tool. Judgements were summarized as “low” or “high” risk of bias or “some concerns” (Higgins et al., 2022).

### 2.5 Statistical analysis

Review Manager 5.4 and Stata 15 were used for data analyses in this review. For continuous data (scores of MMSE and MoCA), mean difference (MD) with 95% confidence intervals (CIs) was calculated. For categorical data (adverse event), risk ratio (RR) and 95% CIs were used to present the effect size. A random-effect meta-analysis model was utilized to calculate the pooled effect size of all outcomes. Meta-analyses were conducted on the end-of-treatment data of the primary outcome measures, MMSE and MoCA, to evaluate the efficacy of CHM on cognitive function among individuals with MCI. Heterogeneity between trials was assessed using the I^2^ test, which was incorporated into the forest plots. Subgroup analyses were conducted based on treatment duration, subtypes of MCI and herbal ingredients, to explore the source of heterogeneity. Several sensitivity analyses, including the use of different effect models, exclusion of studies that were considered to be at a “high risk of bias” in the overall judgment or each domain of the ROB 2, and a one-by-one exclusion approach, were conducted to assess the robustness of the finding. The potential presence of publication bias in the primary outcomes was evaluated by constructing a funnel plot and using Egger’s test when the number of included studies exceeded ten (Sterne et al., 2011).

### 2.6 Certainty of the evidence

The certainty of the evidence for the primary outcome, cognitive function evaluated using validated tools (MMSE and MoCA), was assessed using the Grading of Recommendations Assessment, Development, and Evaluation (GRADE) method. The certainty was categorized as “high”, “moderate”, “low”, or “very low” considering the risk of bias, inconsistency of results, indirectness of evidence, imprecision, and publication bias (Schünemann et al., 2013).

## 3 Results

### 3.1 Results of the search

**FIGURE 1:**
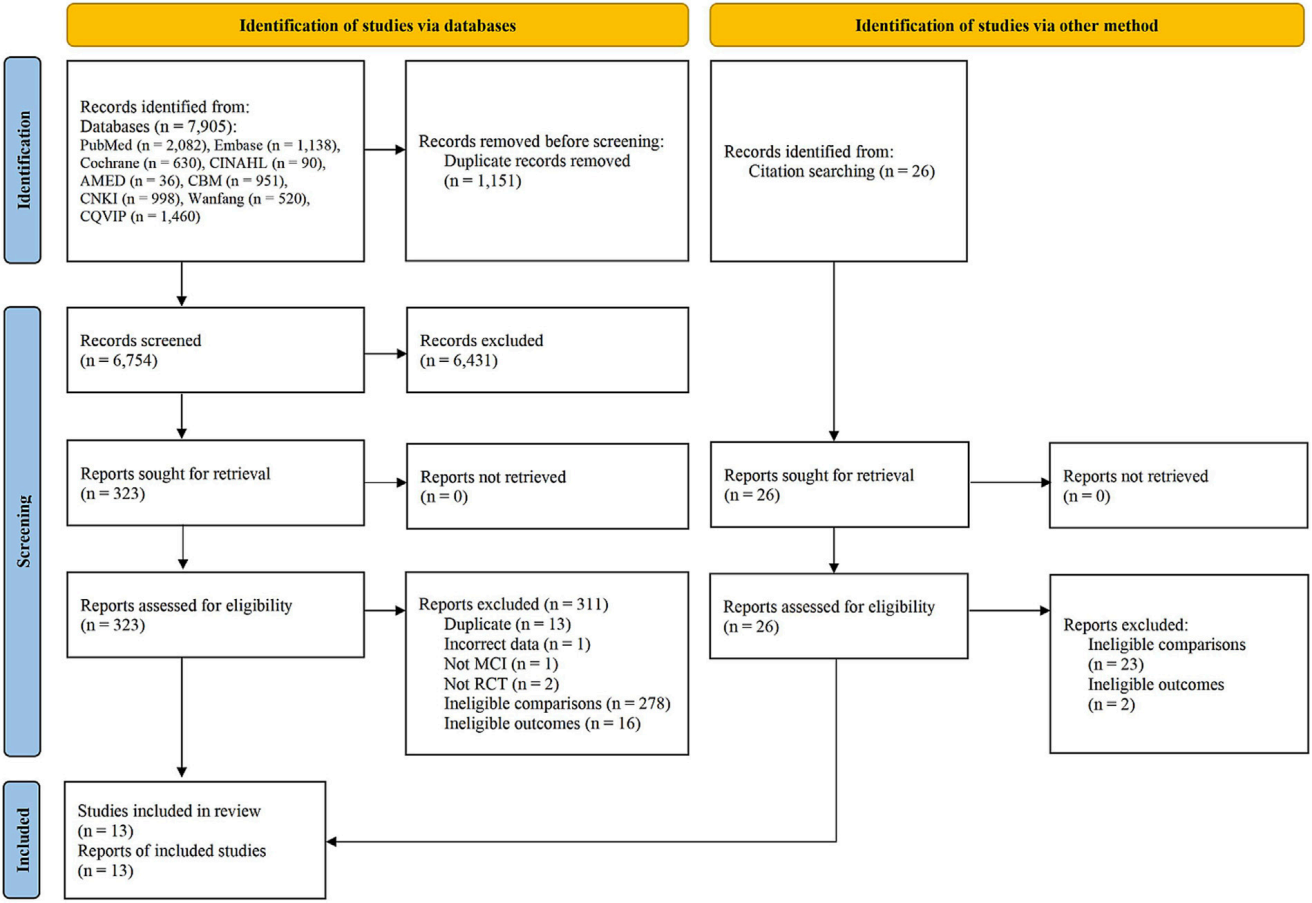
Flowchart of study selection. Abbreviations: AMED, Allied and Complementary Medicine Database; CBM, China Biomedical Literature; CHM, Chinese Herbal Medicine; CINAHL, Cumulative Index to Nursing and Allied Health Literature; CNKI, China National Knowledge Infrastructure database; Cochrane (CENTRAL), Cochrane Central Register of Controlled Trials; CQVIP, Chongqing VIP; Embase, Excerpta Medica Database; MCI, mild cognitive impairment; RCT, randomized controlled trial.

A total of 7,931 records were initially identified by searching the nine databases, of which 7,905 were retrieved for screening; and an additional 26 records were identified by searching reference lists of previously published systematic reviews. After screening, 13 studies were included in this review (Zhou et al., 2007; Wu et al., 2010; Dai et al., 2011; Huang et al., 2013; Su, 2013; Liu, 2014; Shi et al., 2015; Wu, 2016; Zhang et al., 2016; Park et al., 2019; Lu et al., 2020; Chen et al., 2021; Shin et al., 2021). The study search and selection procedure is shown in Figure 1.

### 3.2 Characteristics of included studies

The 13 studies included in this review were published between 2007 and 2021. Five studies were published in English (Zhang et al., 2016; Park et al., 2019; Lu et al., 2020; Chen et al., 2021; Shin et al., 2021) and the other eight studies were published in Chinese (Zhou et al., 2007; Wu et al., 2010; Dai et al., 2011; Huang et al., 2013; Su, 2013; Liu, 2014; Shi et al., 2015; Wu, 2016). Ten trials were conducted in China (Zhou et al., 2007; Wu et al., 2010; Dai et al., 2011; Huang et al., 2013; Su, 2013; Liu, 2014; Shi et al., 2015; Wu, 2016; Zhang et al., 2016; Lu et al., 2020), two in South Korea (Park et al., 2019; Shin et al., 2021), and one involved three centers in Singapore, Vietnam, and the Philippine (Chen et al., 2021). Detailed characteristics of each study are presented in Table 1.

**TABLE 1:** Characteristics of included studies.

Study	Country	Sample size randomized (dropouts)		Main inclusion criteria of the study population	Treatment		Treatment duration/Follow-up duration	Outcome measures
		CHM group	Placebo group		CHM group	Placebo group		
Zhou et al. (2007)	China	42 (2)	37 (2)	MMSE: 21–27, GDS: level 2–3; Petersen criteria for MCI	*Shen yin* oral solution	placebo	12 months/6 months	MMSE, CDT, figure test, AEs
Wu et al. (2010)	China	65 (0)	63 (0)	MMSE: 24–27/illiteracy: 22–25; Petersen criteria for MCI	*Tian tai* No.1capsule	placebo	6 months/1 year	MMSE, Clinic efficacy index, AEs
Dai et al. (2011)	China	25 (0)	25 (0)	GDS: level 2–3 or CDR = 0.5; Petersen criteria for MCI	*Yi zhi jian nao* granule	placebo	16 weeks/1 year	MMSE, MQ
Huang et al. (2013)	China	53 (2)	51 (3)	MMSE: 24–27; CDR = 0.5; Petersen criteria for MCI	*Naokang* II decoction	placebo	2 months/No	MMSE, MoCA, TESS
Su (2013)	China	50 (2)	50 (1)	MMSE: 24–27; CDR = 0.5; Petersen criteria for MCI	*Jian pi tian jing* formula (granule)	placebo	6 months/No	MMSE, MoCA, ADL, AEs
Liu (2014)	China	24 (1)	24 (2)	MMSE: 23–26, MoCA: 20–25; VCIND	*Xiao xu ming* decoction + usual care (control of hypertension, diabetes, hyperlipidemia, antiplatelet therapy performed with indications)	placebo + usual care (control of hypertension, diabetes, hyperlipidemia, antiplatelet therapy performed with indications)	90 days/No	MMSE, MoCA, IDAL, AEs
Shi et al. (2015)	China	35 (0)	35 (0)	MMSE: illiteracy >17, primary school >20, above secondary school >24; GDS: scores 2–3, CDR = 0.5; Chinese expert consensus criteria for MCI	*Yi zhi jian* granule	placebo	90 days/No	MMSE, P300, AEs
Wu (2016)	China	62 (2)	16 (1)	MMSE: 24–30; CDR = 0.5, ADL <16; Petersen criteria for MCI	*Bu shen jian pi hua* tan pill	placebo	12 weeks/No	MMSE, CIBIC-Plus, AEs
Zhang et al. (2016)	China	30 (0)	30 (0)	MMSE ≥24; Petersen criteria for amnestic MCI	*Bu shen* capsule	placebo	24 months/No	MMSE, CAB, brain MRI, AEs
Park et al. (2019)	South Korea	45 (4)	45 (4)	K-MMSE >26; Petersen criteria for MCI	*Panax ginseng* powder	placebo	24 weeks/(Yes, duration unclear)	K-MMSE, K-IADL, SNSB, AEs
Lu et al. (2020)	China	50 (5)	50 (13)	VCIND	*Deng zhan sheng mai* capsule	placebo	6 months/No	MMSE, ADAS-Cog, CAB, brain MRI
Chen et al. (2021)	Singapore, Vietnam, Philippines	57 (3)	46 (1)	mRS <3, VCIND	MLC901 capsule + standard post-stroke care^a^	placebo + standard post-stroke care^a^	24 weeks/No	MoCA, CCT, VF test, ADAS-Cog, CAB, ADCS-ADL, NPI, GDS*, AEs
Shin et al. (2021)	South Korea	17 (1)	16 (2)	GDS = 3, CDR = 0.5, normal K-MMSE; neurologist confirmed amnestic MCI	*Kami-guibi-tang* granule + usual care (control of underlying diseases, such as hypertension, diabetes)	placebo + usual care (control of underlying diseases, such as hypertension, diabetes)	24 weeks/4 weeks	K-MMSE, SNSB, CDR, GDS, Barthel-ADL, K-IADL, GDS*, laboratory test, brain MRI, AEs

Abbreviations: ADAS-Cog, Alzheimer’s Disease Assessment Scale-Cognitive Subscale; ADCS-ADL, Alzheimer’s Disease Cooperative Study Activities of Daily Living; ADL, activities of daily living; AEs, adverse events; Barthel-ADL, Barthel-Activities of Daily Living; CAB, Cognitive Assessment Battery (CAB, in (Chen et al., 2021) comprised of Symbol Digits Modalities Test, Digital Cancellation Test, Visual Memory test, Frontal Assessment Battery; CAB, in (Lu et al., 2020) comprised of Auditory Verbal Learning Test, Rey-Osterrieth Complex Figure-Delay Recall Test, Rey-Osterrieth Complex Figure-Copy, Clock Drawing Test, Digit Span test backward, Stroop Color and Word Test (C-B time), Trail Making Test (B-A time), Symbol Digit Modalities Test, similarity test, Category verbal fluency tests, Boston Naming Test; CAB, in (Zhang et al., 2016) comprised of Auditory Verbal Learning Test, Rey-Osterrieth Complex Figure test (recall), Digit Span test (a sub-test of the Wechsler Adults Intelligence Scale–Chinese revision), Trail Making Test, Symbol Digit Modalities Test, Stroop Color and Word Test (SCWT) (A and B), Rey-Osterrieth Complex Figure-Copy, Clock-Drawing Test, Category verbal fluency tests, Boston Naming Test, Trail Making test-B, Stroop Color Word test -C; CCT, colour trail test; CDR, clinical dementia rating; CDT, clock drawing test; CIBIC-Plus, Clinician Interview Based Impression of Change Plus Care-giver Input; GDS*, geriatric depression scale; GDS, global deterioration scale; IADL, instrumental activities of daily living; K-IADL, korean version of instrumental activities of daily living; K-MMSE, Koeran version Mini-Mental State Examination; MCI, mild cognitive impairment; MMSE, Mini-Mental State Examination; MoCA, montreal cognitive assessment; MQ, memory quotient (Wechsler Memory Scale); MRI, magnetic resonance imaging; mRS, modified Rankin Score; NPI, neuropsychiatric inventory; P300: P300 is an event-related potential used as an tool to assess cognitive function; SNSB, seoul neuropsychological screening battery; TESS, treatment emergent symptom scale; TIA, transient ischemic attack; VCIND, vascular cognitive impairment no dementia; VF, verbal fluency.

A standard post-stroke care included any concomitant medication that the subjects are administered for secondary stroke prevention.

A total of 1,043 participants were enrolled in the 13 studies. The number of participants per trial ranged from 33 to 128, with 80 participants on average. Nine studies reported a total of 51 dropouts, of which 22 were from the CHM groups and 29 were from the placebo groups. Ten trials recruited patients diagnosed with MCI without indicating a specified subtype, and three trials recruited only vascular MCI (i.e., vascular cognitive impairment no dementia) (Liu, 2014; Lu et al., 2020; Chen et al., 2021). Eleven studies reported the baseline scores of MMSE: the mean MMSE score in ten studies ranged from 23 to 27 (Zhou et al., 2007; Wu et al., 2010; Dai et al., 2011; Huang et al., 2013; Su, 2013; Liu, 2014; Wu, 2016; Zhang et al., 2016; Park et al., 2019; Lu et al., 2020; Shin et al., 2021), the other one reported an extremely low mean MMSE score (Shi et al., 2015). Four studies reported the baseline scores of MoCA (Huang et al., 2013; Su, 2013; Liu, 2014; Chen et al., 2021), with the mean scores ranging from 18 to 21. Table 2 presents detailed information on the participants’ baseline characteristics, including age, gender, education level and cognitive score.

**TABLE 2:** Participants’ characteristics of included studies.

Study	Age (mean ± SD), years		Gender (male/female)		Education (mean ± SD), years		Baseline assessment	Baseline cognitive score (mean ± SD)/median (IQR)	
	CHM group	Placebo group	CHM group	Placebo group	CHM group	Placebo group		CHM group	Placebo group
Zhou et al. (2007)	70.93 ± 6.41	68.51 ± 5.93	21/21	20/17	Not reported	Not reported	MMSE	25.93 ± 1.10	25.70 ± 1.18
Wu et al. (2010)	74.26 ± 6.67	74.38 ± 6.22	36/29	41/22	Not reported	Not reported	MMSE	24.69 ± 1.49	24.44 ± 1.62
Dai et al. (2011)	70.50 ± 7.20	69.30 ± 8.10	12/13	10/15	10.28	11.35	MMSE	23.90 ± 2.37	24.10 ± 2.32
Huang et al. (2013)	Data for the whole sample		Data for the whole sample: 57/47		Not reported	Not reported	MMSE	26.07 ± 0.93	26.19 ± 1.12
	Male: 61.54 ± 8.37						MoCA	19.40 ± 3.37	19.71 ± 2.90
	Female: 60.90 ± 9.16								
Su (2013)	82.19 ± 3.43	81.53 ± 3.96	35/13	32/17	Primary school and below (n = 23), secondary school (n = 18), university and above (n = 7)	Primary school and below (n = 28), secondary school (n = 16), university and above (n = 5)	MMSE	23.23 ± 1.64	23.80 ± 1.96
							MoCA	21.15 ± 2.49	20.63 ± 3.24
Liu (2014)	64.65 ± 7.70	65.14 ± 6.46	12/11	13/9	Not reported	Not reported	MMSE	23.87 ± 0.87	23.68 ± 0.78
							MoCA	21.57 ± 1.12	21.36 ± 1.05
Shi et al. (2015)	70.30 ± 18.80	69.80 ± 19.60	21/14	23/12	7.40 ± 3.40	7.20 ± 4.20	MMSE	14.80 ± 3.10	15.10 ± 3.50
Wu (2016)	66.05 ± 9.80	67.47 ± 8.98	26/36	7/9	11.27 ± 3.27	11.10 ± 3.42	MMSE	26.38 ± 2.08	25.80 ± 3.08
Zhang et al. (2016)	66.00 ± 6.86	63.33 ± 6.65	16/14	12/18	10.37 ± 3.41	10.33 ± 3.53	MMSE	26.00 ± 2.05	26.67 ± 1.45
Park et al. (2019)	61.80 ± 6.90	62.60 ± 6.30	15/30	15/30	Not reported	Not reported	K-MMSE	27.85 ± 1.11	27.73 ± 1.07
Lu et al. (2020)	65.56 ± 8.32	65.97 ± 7.89	22/23	20/17	11.56 ± 3.93	11.55 ± 4.62	MMSE	25.53 ± 3.70	26.27 ± 4.05
(Chen et al., 2021)	69.40 ± 8.20	67.20 ± 8.60	32/25	31/15	Not reported	Not reported	MoCA	19.20 ± 5.10	18.00 ± 5.10
Shin et al. (2021)	70.20 ± 7.60	70.10 ± 6.40	10/6	7/7	12.2 ± 3.60	12.30 ± 4.70	K-MMSE	28.5 (26.3–29.0)	26.0 (24.8–28.3)
								^a^27.71 ± 2.34	^a^26.48 ± 2.99

Abbreviations: CHM, chinese herbal medicine; IQR, interquartile range; K-MMSE, Korean version Mini-Mental State Examination; MMSE, Mini-Mental State Examination; MoCA, montreal cognitive assessment; SD, standard deviation.

^a^ The mean and SD, value was estimated using the Box-Cox method (McGrath et al., 2020).

All 13 included studies compared CHM with placebo. The treatment duration varied from two to 24 months, with eight out of 13 studies having a duration of 6 months or more. Among the 13 studies, four conducted follow-up assessments after the treatment phase, ranging from 1 month to 1 year (Zhou et al., 2007; Wu et al., 2010; Dai et al., 2011; Shin et al., 2021). One study mentioned that participants were followed-up after the treatment phase, but did not specify the duration (Park et al., 2019). The remaining eight studies did not include a follow-up phase after the treatment.

Twelve studies evaluated the treatment effects using MMSE (Zhou et al., 2007; Wu et al., 2010; Dai et al., 2011; Huang et al., 2013; Su, 2013; Liu, 2014; Shi et al., 2015; Wu, 2016; Zhang et al., 2016; Park et al., 2019; Lu et al., 2020; Shin et al., 2021), and three studies reported data on MoCA (Huang et al., 2013; Su, 2013; Liu, 2014). Eleven studies clearly outlined in the methods section that AEs would be monitored during the study period, and reported the outcome of AEs in the published articles (Zhou et al., 2007; Wu et al., 2010; Huang et al., 2013; Su, 2013; Liu, 2014; Shi et al., 2015; Wu, 2016; Zhang et al., 2016; Park et al., 2019; Chen et al., 2021; Shin et al., 2021). The remaining two studies reported information on AEs in the results/discussion sections without stating this in the methods section (Dai et al., 2011; Lu et al., 2020).

### 3.3 Quality control and ingredients of CHM/placebo preparations

Except four studies (Huang et al., 2013; Liu, 2014; Shi et al., 2015; Zhang et al., 2016), the other nine studies identified the pharmaceutical manufacturer. Nonetheless, only one study explicitly stated that both the CHM preparation and the placebo were produced by the same manufacturer, utilizing standardized methods in accordance with Good Manufacturing Practice guidelines (Shin et al., 2021). The remaining studies, however, did not specify the quality control methods implemented for the CHM or placebo preparations (Supplementary Table S2).

Out of the 13 included studies, two used the form of decoction for CHM administration (Huang et al., 2013; Liu, 2014). The other 11 studies utilized more convenient format of CHM preparation, they are: capsules (Wu et al., 2010; Zhang et al., 2016; Lu et al., 2020; Chen et al., 2021), granules (Dai et al., 2011; Su, 2013; Shi et al., 2015; Shin et al., 2021), powder (Park et al., 2019), pills (Wu, 2016) and oral solution (Zhou et al., 2007). Thirteen unique CHM formulae were identified from the 13 included studies. Twelve studies detailed the herbal ingredients of the CHM formulae, and the other one study did not provide such information (Wu, 2016). A total of 50 herbs were used in these trials. Although the formulae were diverse across trials, some herbs were frequently used by most of the studies, with the most common herbs being *Panax ginseng* C.A.Mey. (*Ren shen*), *Acorus calamus* var. *angustatus* Besser (*Shi chang pu*), *Polygala tenuifolia* Willd. (*Yuan zhi*). Supplementary Table S3 presents the top 15 most frequently used herbs.

All 13 included studies used a placebo CHM as the control, but the ingredients used to prepare the placebo varied across the trials. Seven trials prepared placebo only containing inactive substances such as starch, food coloring and bittering agent (Zhou et al., 2007; Wu et al., 2010; Shi et al., 2015; Park et al., 2019; Lu et al., 2020; Chen et al., 2021; Shin et al., 2021). In one study, the placebo comprised a bittering agent, food coloring, and stir-fried Medicated leaven (*Shen qu*) (Huang et al., 2013). It is worth noting that Medicated leaven is a fermented product by mixing flour with other Chinese medicines (including *Artemisia annua* Pall., *Xanthium sibiricum* Patrin ex Widder, *Polygonum hydropiper* L., *Vigna angularis* (Willd.) Ohwi & H. Ohashi, *Prunus armeniaca* L.), which has been commonly used to treat gastrointestinal diseases in Chinese medicine (Fu et al., 2020). Another study stated that the placebo consisted of a 10% dose of the CHM decoction used in the experimental group (Liu, 2014). Four studies did not provide detailed information about the preparation of the placebo. However, three out of these four studies indicated that the appearance, smell and taste of the placebos were the same as the CHM in the experimental groups (Dai et al., 2011; Su, 2013; Zhang et al., 2016), while the remaining one study mentioned that the control group received a mimetic agent with a corresponding dosage to the CHM (Wu, 2016). Detailed information on quality control and ingredients of CHM/placebo preparations are presented in Supplementary Table S2.

### 3.4 Risk of bias assessment

We used the Cochrane’s Risk of Bias (RoB) 2 tool to assess the risk of bias of included studies based on two outcome measures: MMSE and MoCA. The results are merged in Supplementary Figure S1 since there is no difference between the assessments based on these two outcomes. In our assessment, an intention-to-treat analysis model was used. In addition to the published results articles, the trial registry records related to four trials (Zhang et al., 2016; Lu et al., 2020; Chen et al., 2021; Shin et al., 2021) and one published protocol related to one trial (Chen et al., 2021) were also checked to inform the RoB assessment.

For overall RoB, 10 studies (76.9%) were assessed as “some concerns”, one study (Shin et al., 2021) is “low risk of bias” and two studies are “high risk” (Lu et al., 2020; Chen et al., 2021). In terms of randomization process, six studies were judged as “some concerns” because they did not provide information on the generation of allocation sequence and sequence concealment (Zhou et al., 2007; Wu et al., 2010; Dai et al., 2011; Huang et al., 2013; Shi et al., 2015; Park et al., 2019), the others were assessed as “low risk of bias” for this domain. For the domain of “deviations from the intended interventions”, five studies were assessed as “some concerns” because they did not apply an intention-to-treat analysis to deal with missing data (Huang et al., 2013; Su, 2013; Liu, 2014; Wu, 2016; Chen et al., 2021), one study was “high risk of bias” for this domain due to a high dropout rate (18%) without appropriate analysis method (Lu et al., 2020); the remaining seven studies were “low risk of bias” since there was no dropout cases. The Lu 2020 study (Lu et al., 2020) was also given a “high risk of bias” judgement for “missing outcome data”. In terms of the outcome measurements, all 13 studies were assessed as “low risk of bias” since both MMSE and MoCA consist of a series of questions with clearly defined scoring criteria, which were unlikely to be influenced by knowing which intervention was received. For the “selection of the reported result”, one study was assessed as “high risk of bias” because detailed data on a pre-specified outcome (MMSE) was not reported (Chen et al., 2021); 10 studies were rated as “some concerns”, due to a lack of pre-registered trial protocols or insufficient information on outcome measurements and statistical analyses plans provided in the trial registration; the remaining two studies were rated as “low risk of bias” (Park et al., 2019; Shin et al., 2021).

### 3.5 MMSE

#### 3.5.1 Overall effects

**FIGURE 2:**
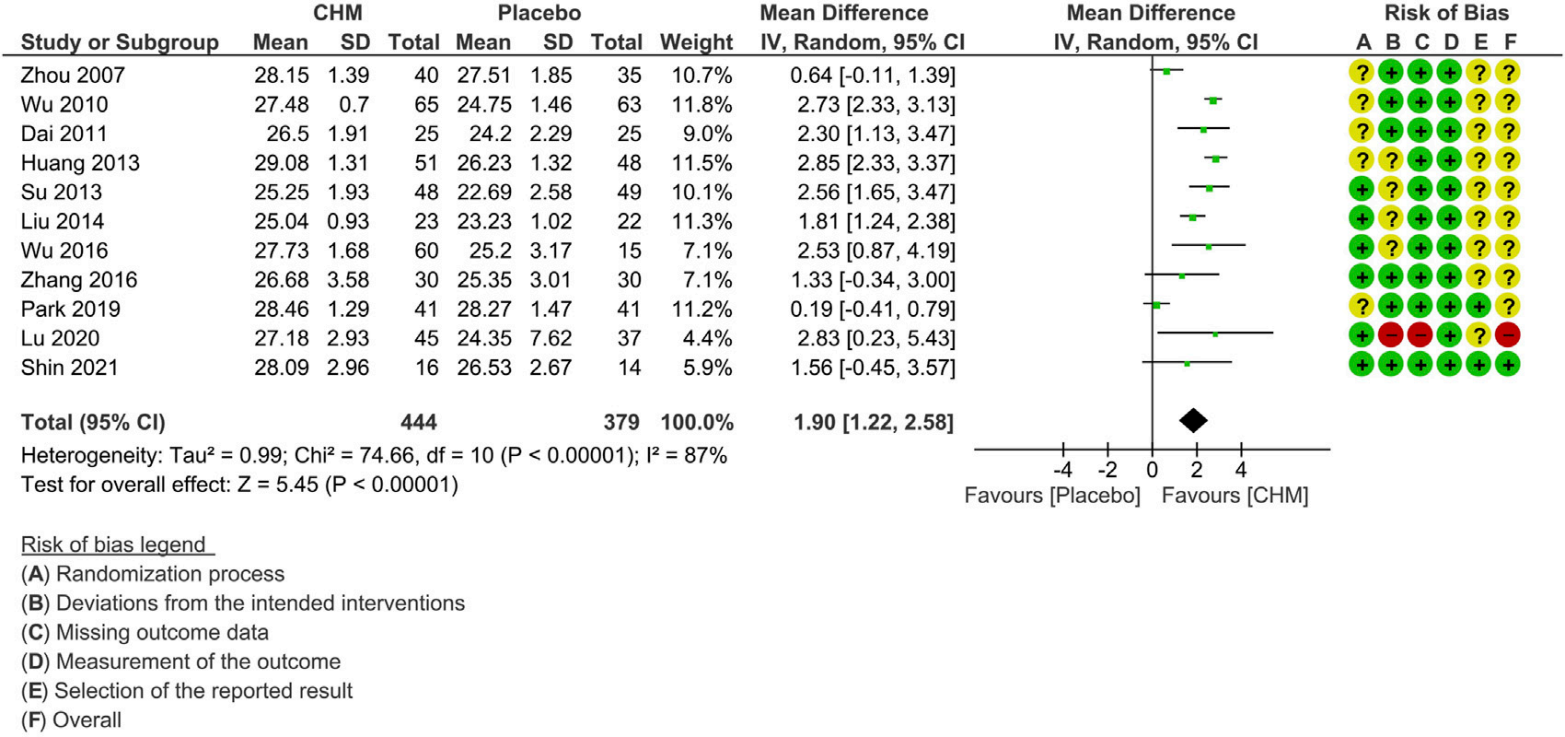
Forest plot for the outcome of MMSE at the end of treatment. Abbreviation: CHM, Chinese herbal medicine.

All except one study (Chen et al., 2021) reported MMSE scores at the end of treatment phase. One study was excluded from the meta-analysis due to a notably low baseline MMSE score, which deviated from the average level characteristic of MCI (Shi et al., 2015). We contacted the authors for clarification but did not receive any response. The pooled results on 11 studies revealed a significant improvement in MMSE among the CHM group compared with the placebo group (MD: 1.90 [1.22, 2.58], I^2^ = 87%, 11 studies, 823 participants) (Figure 2). Since substantial statistical heterogeneity was observed (I^2^ = 87%, *p* < 0.00001), subgroup analyses were conducted to explore potential sources of heterogeneity.

#### 3.5.2 Subgroup analyses

The subgroup analysis based on the subtypes of MCI included nine studies that did not specify such information (Zhou et al., 2007; Wu et al., 2010; Dai et al., 2011; Huang et al., 2013; Su, 2013; Wu, 2016; Zhang et al., 2016; Park et al., 2019; Shin et al., 2021) and two studies on vascular MCI (Liu, 2014; Lu et al., 2020).

**FIGURE 3:**
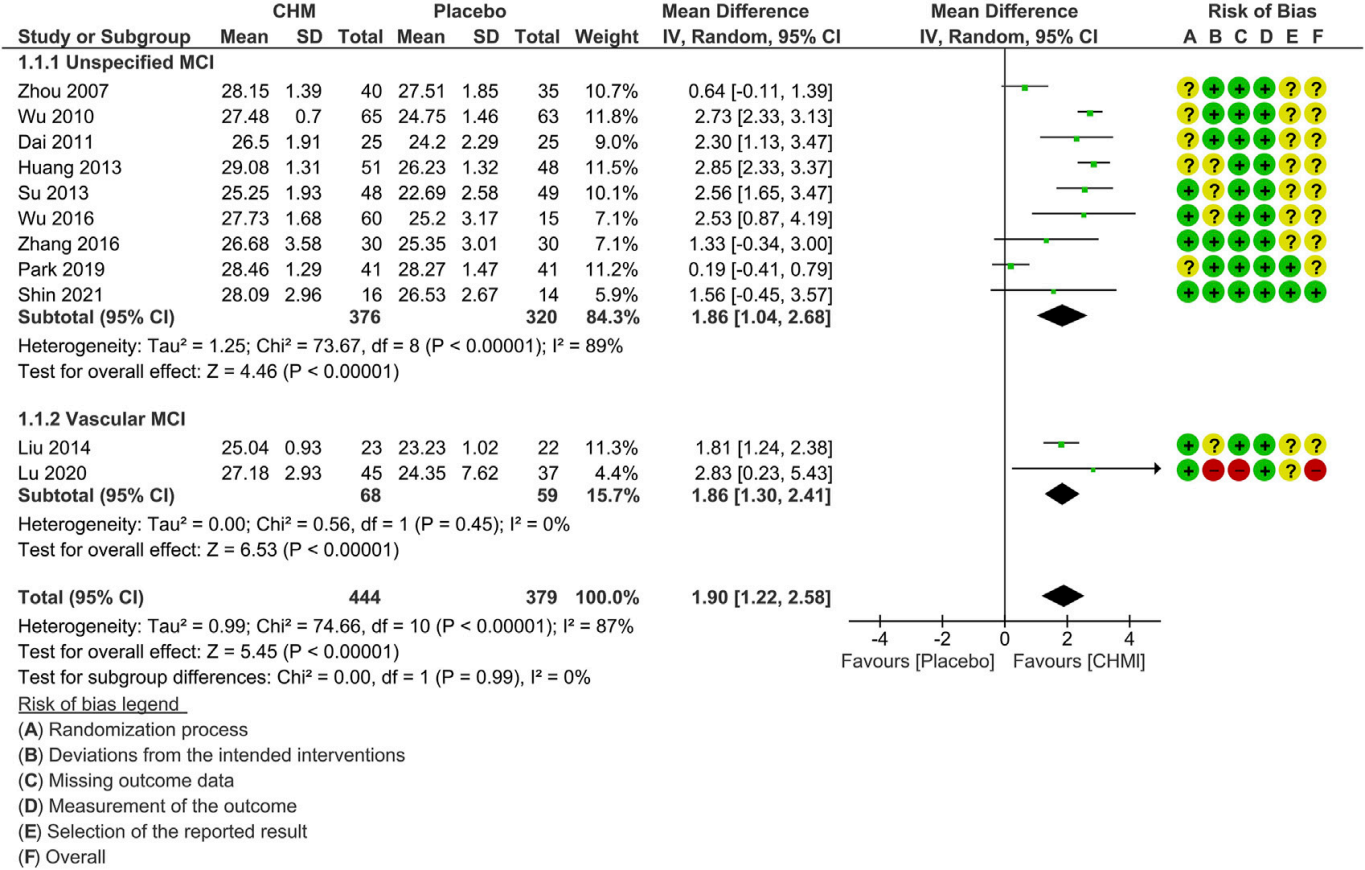
Forest plot for the outcome of MMSE at the end of treatment (subgroup analysis based on the subtypes of MCI). Abbreviations: CHM, Chinese herbal medicine; MCI, mild cognitive impairment.

The results showed that CHM treatment provided similar benefits in both subgroups in terms of MMSE scores. However, the subgroup with unspecified subtypes exhibited higher heterogeneity (MD: 1.86 [1.04, 2.68]; I^2^ = 89%, 9 studies, 696 participants), while the vascular MCI subgroup demonstrated no significant heterogeneity (MD: 1.86 [1.30, 2.41]; I^2^ = 0%, 2 studies, 127 participants) (Figure 3).

**FIGURE 4:**
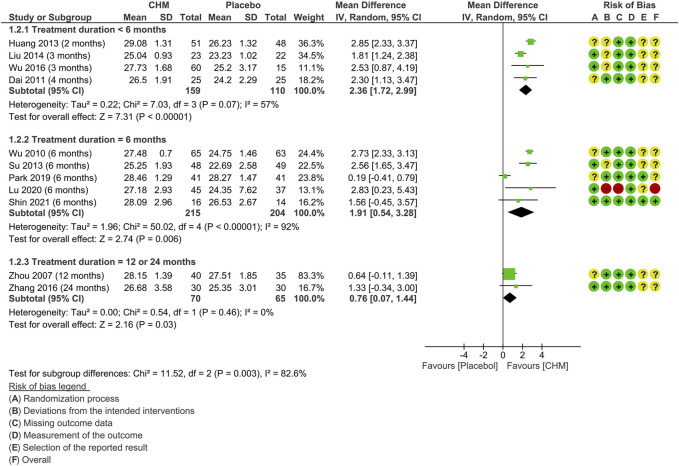
Forest plot for the outcome of MMSE at the end of treatment (subgroup analysis based on the treatment duration). Abbreviation**:** CHM, Chinese herbal medicine.

Subgroup analysis on MMSE according to the treatment duration was also conducted (Figure 4). The test for subgroup differences was significant (*p* = 0.003). The results indicated that there is a gradual decline in the relative benefits of CHM in improving MMSE scores along with the treatment duration prolonged: two to 4 months (MD: 2.36 [1.72, 2.99]; I^2^ = 57%, 4 studies, 269 participants) (Dai et al., 2011; Huang et al., 2013; Liu, 2014; Wu, 2016); 6 months (MD: 1.91 [0.54, 3.28], I^2^ = 92%, 5 studies, 419 participants) (Wu et al., 2010; Su, 2013; Park et al., 2019; Lu et al., 2020; Shin et al., 2021); 12 or 24 months (MD: 0.76 [0.07, 1.14], I^2^ = 0%, 2 studies, 135 participants) (Zhou et al., 2007; Zhang et al., 2016).

Multiple subgroup analyses were conducted based on the presence of the most frequently used herbs in the CHM prescription (Table 3). The subgroup analyses revealed that the studies utilizing *P. ginseng* C.A.Mey. (Wu et al., 2010; Su, 2013; Liu, 2014; Park et al., 2019; Lu et al., 2020; Shin et al., 2021), *A. calamus* var. *angustatus* Besser (Dai et al., 2011; Huang et al., 2013), *P. tenuifolia* Willd. (Dai et al., 2011; Huang et al., 2013; Shin et al., 2021) demonstrated greater improvements in MMSE scores compared to non-use, however, only the analysis on *A. calamus* var. *angustatus* Besser detected a statistically significant between-group difference. It is worth noting that, *A. calamus* var. *angustatus* Besser and *P. tenuifolia* Willd. are often used as a pair of herbs in Chinese medicine clinical practice, carrying the function of refreshing the mind and enhancing cognitive function synergistically (Luo et al., 2020). Subgroup analysis also confirmed that the studies used both *A. calamus* var. *angustatus* Besser and *P. tenuifolia* Willd. achieved a statistically significant better effects than those not using these two herbs (Dai et al., 2011; Huang et al., 2013). It should be pointed out that, due to the limited number of studies, it is not feasible to conduct further analysis, such as meta-regression analysis, to explore the potential interaction between different variables.

**TABLE 3:** Subgroup analysis of MMSE at the end of treatment (with or without top three frequently used herbs^a^).

Subgroups	No. of studies	No. of participants	MD [95% CI]	I^2^%
1) With and without *Ren shen* (Test for subgroup difference: *p* = 0.94)				
With *Ren shen*	6	464	1.88 [0.86, 2.89]	90
Wthout *Ren shen*	4	284	1.81 [0.55, 3.07]	87
2) With and without *Shi chang pu* (Test for subgroup difference: *p* = 0.03)				
With *Shi chang pu*	2	149	2.76 [2.29, 3.23]	0
Without *Shi chang pu*	8	599	1.64 [0.78, 2.50]	89
3) With and without *Yuan zhi* (Test for subgroup difference: *p* = 0.05)				
With *Yuan zhi*	3	179	2.70 [2.23, 3.16]	0
Without *Yuan zhi*	7	569	1.65 [0.73, 2.57]	90
4) With and without *Shi chang pu* & *Yuan zhi* (Test for subgroup difference: *p* = 0.03)				
With *Shi chang pu* & *Yuan zhi*	2	149	2.76 [2.29, 3.23]	0
Without *Shi chang pu* & *Yuan zhi*	8	599	1.64 [0.78, 2.50]	89

Abbreviations: CI, confidence interval; I^2^: index of heterogeneity; IV, inverse variance; MD, mean difference.

^a^ Top three frequently used herbs: *Panax ginseng* C.A.Mey. (*Ren shen*), *Acorus calamus* var. *angustatus* Besser (*Shi chang pu*), *Polygala tenuifolia* Willd. (*Yuan zhi*).

#### 3.5.3 Sensitivity analysis

Sensitivity analysis was performed to investigate the robustness of the treatment effects based on MMSE (Table 4). The between-group difference did not change significantly when switching from a random-effect model to a fixed-effect model. Similarly, when selecting the studies at “low risk of bias” or “some concerns” in overall judgement on RoB, at “low risk of bias” for other domains (randomization process, deviations from the intended interventions, missing outcome data, measurement of the outcome), sensitivity analyses showed similar results to the overall effect estimate of all studies. However, when selecting “low risk of bias” in overall (Shin et al., 2021) or for the reported outcome (Park et al., 2019; Shin et al., 2021), the sensitivity analysis results showed that there might be no difference between the CHM and placebo group.

**TABLE 4:** Sensitivity analysis of the treatment effect for the MMSE outcome.

Sensitivity analysis	No. of studies	No. of participants	Statistical method	MD [95% CI]	I^2^%
All studies	11	823	IV, Random effect	1.90 [1.22, 2.58]	87
All studies	11	823	IV, Fixed effect	2.03 [1.81, 2.25]	87
Low risk or some concerns in overall judgement	10	741	IV, Random effect	1.86 [1.15, 2.56]	88
Low risk in overall judgement	1	30	IV, Random effect	1.56 [-0.45, 3.57]	N/A
Low risk in randomization process	6	389	IV, Random effect	2.01 [1.58, 2.44]	0
Low risk in deviations from the intended interventions	7	425	IV, Random effect	1.45 [0.29, 2.61]	92
Low risk in missing outcome data	10	741	IV, Random effect	1.86 [1.15, 2.56]	88
Low risk in measurement of the outcome	11	823	IV, Random effect	1.90 [1.22, 2.58]	87
Low risk in selection of the reported outcome	2	112	IV, Random effect	0.52 [-0.63, 1.68]	39

Abbreviations: CI, confidence interval; I^2^: index of heterogeneity; IV, inverse variance; MD, mean difference; N/A: not applicable.

In order to detect the influence of a single study on the overall pooled estimate, we also conducted sensitivity analysis by removing one study at a time. The findings showed that the removal of any of these 11 RCTs did not lead to a significant change in the overall effect estimate (Supplementary Figure S2).

Overall, sensitivity analysis indicated that the results were robust regardless of the inclusion or exclusion of any individual studies.

#### 3.5.4 Publication bias

**FIGURE 5:**
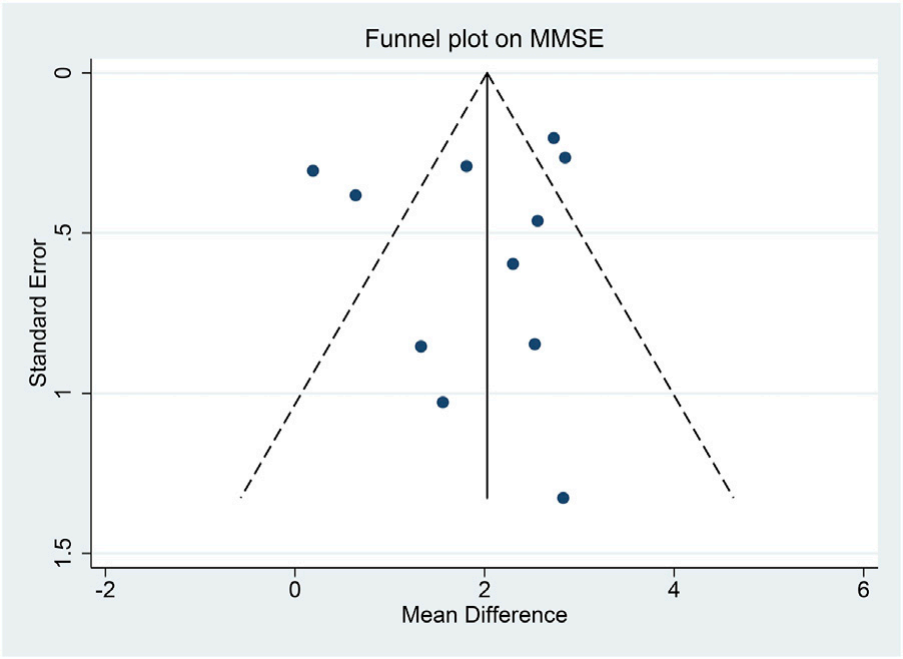
Funnel plot of studies reporting MMSE at the end of treatment.

In order to explore publication bias for the end-of-treatment outcome of MMSE, a funnel plot and Egger’s test were conducted. The funnel plot was symmetrical, and no significant difference was found from Egger’s test (*p* = 0.625), indicating that publication bias was improbable (Figure 5).

### 3.6 MoCA

**FIGURE 6:**
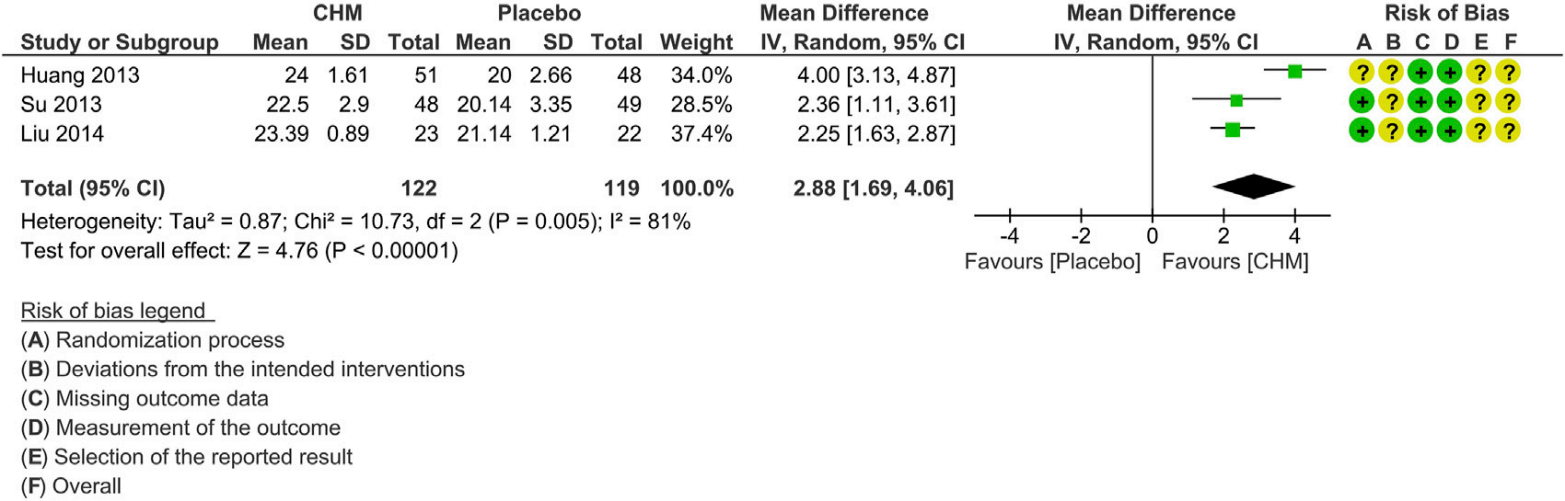
Forest plot for the outcome of MoCA at the end of treatment. Abbreviation: CHM, Chinese herbal medicine.

Three studies reported MoCA scores at the end of treatment (Huang et al., 2013; Su, 2013; Liu, 2014). Meta-analysis shows that the CHM group achieved superior effects in improving the MoCA score compared to the placebo group (MD: 2.88 [1.69, 4.06], I^2^ = 81%, 3 studies, 241 participants) (Figure 6). Subgroup analysis, sensitivity analysis, and publication bias evaluation were not feasible due to the small number of studies reporting this outcome.

### 3.7 Adverse events

Twelve studies mentioned AE information: four studies reported no AEs during the treatment phase (Wu et al., 2010; Dai et al., 2011; Su, 2013; Shi et al., 2015) and eight studies reported AEs occurred (Zhou et al., 2007; Huang et al., 2013; Liu, 2014; Wu, 2016; Zhang et al., 2016; Park et al., 2019; Chen et al., 2021; Shin et al., 2021). Among the eight studies reporting AEs, one study mentioned that no statistical difference was detected between the CHM and placebo group in terms of the Treatment Emergent Symptom Scale score (Huang et al., 2013). However, the exact number of participants who experienced the AEs was not reported (Huang et al., 2013). Therefore, the remaining 11 studies were included in the meta-analysis for AEs, with varied adverse events observed in 76 participants (9.06%). There is no statistically significant difference between the CHM and placebo groups in terms of the number of participants who reported AEs (RR: 1.31 [0.92, 1.87], I^2^ = 0%, 11 studies, 839 participants) (Supplementary Figure S3). Notably, gastrointestinal symptoms like constipation, nausea, and vomiting emerged as the predominant manifestations among all the reported AEs. One study reported that one case in the treatment group dropped out from the study due to gastrointestinal discomfort (Liu, 2014). One study did not report the safety outcome but mentioned that of the 18 patients (CHM and placebo groups) who dropped out of the study, none were due to AEs (Lu et al., 2020).

As for the severity of the AE, two studies mentioned that no serious adverse events (SAEs) were observed in either the CHM groups or the placebo groups (Zhou et al., 2007; Liu, 2014). One study reported that, according to the criteria for SAEs which encompassed factors such as death, life-threatening situations, prolonged hospitalization, incapacity, or important medical events, 13 participants (22.8%) experienced SAEs in the CHM group while 6 participants (13.0%) experienced SAEs in the placebo group (Chen et al., 2021). However, there was no significant difference between the CHM and placebo group in terms of the proportion of participants experiencing SAE (Chen et al., 2021).

Regarding the association of AEs with the study interventions, three studies found no identified connection between these AEs and the administered interventions (Su, 2013; Park et al., 2019; Shin et al., 2021). One study noted that a total of six AEs were possibly linked to the administration of the CHM intervention, including dry throat, dizziness, hematoma in the right thigh, vomiting, neuropathic pain, and sleepiness (Chen et al., 2021). Supplementary Table S4 details the AEs reported in the included studies.

### 3.8 Certainty of the evidence

The GRADE assessments for MMSE and MoCA were downgraded by one level due to inconsistency. Additionally, due to the limited sample size, the certainty of the evidence for MoCA was further downgraded by one level for imprecision. Overall, the evidence certainty for MMSE was evaluated as “moderate”, while it was “low” for MoCA (Supplementary Table S5).

## 4 Discussion

### 4.1 Summary of results

This systematic review synthesized 13 RCTs that compared CHM with placebo to evaluate the efficacy and safety of CHM for the treatment of MCI. The meta-analyses on post-treatment MMSE and MoCA scores indicated that, the use of CHM led to significant improvement in MCI patients’ cognitive function compared to placebo.

All except one study reported the outcome of MMSE. The overall effect estimate was found to be robust, and no significant publication bias was detected. However, substantial heterogeneity was observed among the studies. The evidence regarding the outcome on MMSE was assessed with moderate certainty, while it was “low” for MoCA. Subgroup analyses were conducted according to the subtypes of MCI, treatment duration and different CHM herbal ingredients. The results indicated that CHM is beneficial for MCI, either vascular MCI or unclassified MCI. Subgroup analyses also revealed a trend suggesting that the treatment effects of CHM diminish with longer treatment durations. Additionally, herbs include *P. ginseng* C.A.Mey., *A. calamus* var. *angustatus* Besser, *P. tenuifolia* Willd. May enhance the therapeutic effects of CHM. Regarding the safety of CHM, gastrointestinal symptoms have been identified as the most commonly reported AEs. None of the included studies identified an association between the administration of CHM and a higher risk of AEs. The comprehensive meta-analysis on AEs revealed no significant difference between the CHM and placebo groups. While the available evidence suggests that CHM is generally safe, caution is advised due to the lack of AE reporting or detailed information in some instances.

### 4.2 Comparing this review with prior research in the field

Previous reviews have shown that CHM may improve cognitive functions in patients with MCI (Dong et al., 2016; Dong et al., 2019; Wang et al., 2021; Liang et al., 2022), which is consistent with the results of our study. However, there were some differences in the inclusion criteria between our review and previous reviews.

In our study, we aimed to reflect the real-world clinical practice by including participants with all types of MCI, while previous systematic reviews had a narrow focus on only one specific subtype (Dong et al., 2016; Dong et al., 2019). It is important to note that the etiology and pathogenesis of MCI are highly complex (DeCarli, 2003; Mufson et al., 2012; Jongsiriyanyong and Limpawattana, 2018). Furthermore, MCI typically occurs in older adults (Petersen et al., 2018), and there is a high prevalence of overlapping neuropathology in this population (Brenowitz et al., 2017). Therefore, it can be challenging to distinguish a single or pure pathological subtype of cognitive impairment, even with autopsy (Brenowitz et al., 2017). Based on this background, there is a need to evaluate the effects of CHM on all types of MCI.

As for the intervention, in contrast to the latest systematic review which focused solely on Chinese patent medicine (Liang et al., 2022), our review included all types of CHM interventions to provide more comprehensive and representative evidence for real-world clinical practice.

In addition, we prioritize randomized placebo-controlled trial as the optimal study design to assess the efficacy of CHM, as they provide the highest level of evidence (Burns et al., 2011), and the use of indistinguishable placebos is the most effective method for determining the pure biological effect of an intervention in experimental settings (Cummings et al., 2013). Whilst previous reviews explored the effects of CHM using various types of comparisons (Dong et al., 2016; Dong et al., 2019; Wang et al., 2021; Liang et al., 2022). In comparison to previous reviews, our review includes a larger number of original studies from a more diverse range of countries, thereby providing more comprehensive evidence.

### 4.3 Pharmacological action of frequently used herbs

*Panax ginseng* C.A.Mey., *A. calamus* var. *angustatus* Besser, and *P. tenuifolia* Willd. Were the three most commonly used herbs in the included studies. Moreover, the inclusion of *P. ginseng* C.A.Mey., *A. calamus* var. *angustatus* Besser, and *P. tenuifolia* Willd. appears to enhance the therapeutic effects. These three herbs have been traditionally used in East Asia for its potential cognitive improving effects.

Preclinical studies suggest that the active compounds or extracts from these three herbs—*P. ginseng* C.A.Mey., *A. calamus* var. *angustatus* Besser, and *P. tenuifolia* Willd. Can enhance cognitive function across different experimental models of cognitive impairment. The potential underlying mechanisms include antioxidant, anti-apoptosis, anti-neurotoxicity, anti-cytotoxicity and anti-inflammatory effects, mitigation of Alzheimer’s disease-related pathology, synaptic protection, and the upregulation of neuronal cells through various signaling pathways (Li et al., 2020; Liang et al., 2021; Zhang et al., 2023).

Additionally, these three herbs are traditionally valued for their common function in soothing the heart and calming the mind, indicated for treating symptoms like forgetfulness and sleep disturbances (Chinese Pharmacopoeia Commission, 2020). Emerging research has demonstrated a strong association between sleep disturbances and cognitive decline (Naismith et al., 2010; Suh et al., 2018; Ma et al., 2020). Experimental studies have indicated that ginsenosides, the active chemical compounds in *P. ginseng* C.A.Mey. May reverse memory deficits in animal models suffering from sleep deprivation-induced memory impairment (Lu et al., 2017; Lu et al., 2018). Additionally, active compounds from these three herbs have demonstrated sedative and hypnotic effects, improving sleep in animal studies, which aligns with their traditional use (Cao et al., 2016; Radhakrishnan et al., 2017; Xu et al., 2019; Shao et al., 2020). Details information on the pharmacological effects and potential mechanisms of action is presented in Table 5.

**TABLE 5:** Pharmacological action of herbs with high frequency of use and significant associations.

Herb name in *Pin yin^a^*	Plant names^b^	Preparation	Chemical composition	Subject	Pharmacological effects	Mechanisms of action	References
*Ren shen*	*Panax ginseng* C.A.Mey	Compound	Ginsenoside Rg1	AD mice (transgenic mice, aged mice, ovariectomy plus intracranial injections of D-galactose, hippocampus injury, chronic stress, ovariectomy, injection of okamoto acid, quinolinic acid, the Aβ1-42 and Aβ25-35, dexamethasone, D-galactose, scopolamine)	Antioxidant	SOD ↑, GSH-PX ↑	Liang et al. (2021)
						ROS ↓, MDA ↓	
					Anti-inflammatory	TNF-α ↓, IL-1β ↓, IL-6 ↓, IL-18 ↓, caspase 1 ↓, caspase 5 ↓	
					Upregulation of nerve cells	NSC senescence ↓, cell apoptosis ↓, NSC number ↑, new nerve cells ↑	
					Synapse protection	Ach ↑, BDNF ↑, multiple synaptic proteins ↑	
					Amelioration of AD-related pathology	APP ↓, Tau ↓, Aβ ↓	
		Compound	Ginsenoside Rg2	Vascular dementia rat model	Anti-apoptosis	pro-apoptotic factors BAX and P53 ↓	Zhang et al. (2008)
						anti-apoptotic BCL-2 and HSP70 ↑	
		*Panax ginseng* extract in concentrated form	Ginsenoside (G)-Rb1, G-Rb2, G-Rc, G-Rd, G-Re, G-Rf, G-Rg1, G-Rg2, G-Rg3	Vascular dementia rat model	Anti-apoptosis	neuronal density ↑, VEGF and bFGF protein expression ↑, number of glial fibrillary acidic protein-immunoreactive cells ↓, BCl-2 ↑, BAX protein ↓	Zhu et al. (2018)
					Neuroprotective effect		
		Compound	Ginsenoside Rh1	Sleep deprivation-induced mouse memory impairment model	Nootropic effects	Regulating oxidative stress levels in the cortex and hippocampus	Lu et al. (2017)
					Prevent sleep deprivation-induced memory impairment		
		Compound	Ginsenoside Rh2	Sleep deprivation-induced cognitive deficit mice	Reverse spatial and non-spatial memory impairments induced by sleep deprivation	Attenuating oxidative stress	Lu et al. (2018)
		Compound	Ginsenoside Rg1	Rat model	Sleep-promoting (prolong sleep time and degrades sustainability of wakefulness)	Modulating the noradrenergic system in the locus coeruleus and serotonergic system in the dorsal raphe nucleus	Xu et al. (2019)
		Compound	Ginsenoside Rg5, Ginsenoside Rk1	Rodent model	Sedative and hypnotic effects	Mediating the GABA/serotonin/glutamate nervous system	Shao et al. (2020)
*Shi chang pu*	*Acorus calamus* var. *angustatus* Besser (synonyms: *Acorus tatarinowii* Schott, *Acorus gramineus* var. *Crassispadix* Lingelsh.)	Extract (water)/Extract (acetate)/Defatted decoction	*α*-asarone *ß*-asarone essential oil	Cognitive impairment mouse/rat models (cognitive impairment models were induced by lead, noise stress, LPS, Aβ1-42, D-gal plus AlCl3, scopolamine, ethanol, sodium nitrite, corticosterone, Ibotenic acid, chronic restraint stress, pentobarbital sodium, D-galactose, AlCl3, streptozotocin, pent ylenetet razol, NaNO2)	Anti-apoptosis	SOD ↑, CAT ↑, GSH-PX ↑, MDA ↓, HIF-1 ↓	Li et al. (2020)
					Stimulating cholinergic system	AChE ↓, Ach ↑	
					Anti-apoptosis	BCL-2↑, BAX ↓, caspase 3 ↓, JNK ↓	
					Anti-inflammatory	TNF-α ↓, IL-1β ↓	
					Anti-neurotoxicity	APP ↓, Tau ↓	
					Anti-cytotoxicity	NOS ↓, NO ↓	
					Regulating synaptic plasticity	Dendritic spine density ↑, Synaptic loss ↓	
		Extract (ethanol: water (1:1))	asarone	AD mice (scopolamine-induced AD)	Activating the cholinergic system	AChE ↓, GSH ↑, SOD ↑, Nitrite level ↓	Malik et al. (2023)
					Antioxidant		
					Neuroprotective effect		
		Compound	β-asarone	*In vitro* (Aβ1-42 induced PC12 cell model of AD)	Protective effects against AD (the formation and damage of Aβ1-42)	Promote autophagy	Wang et al. (2019)
						Inhibit Aβ	
		Compound	β-asarone	Aβ induced AD rat model	Antioxidant	SOD ↑, GPX ↑	Saki et al. (2020)
					Neuroprotective effect		
		Extract	volatile oil from *Acorus gramineus*	AD mice (Aβ1-42 injected mice)	Induce the regeneration of hippocampal neurons; promote the growth of hippocampal neurons and the clearance of Aβ	BDNF ↑, tyrosine protein kinase B ↑, neurotrophin-3 expression ↑	Gao et al. (2019)
		Compound	*α*-asarone	Sleep deprivation rat model	Improve the quality of sleep	Minimum variation between hypothalamic temperature and body temperature, enhanced the association between NREM sleep about duration and hypothalamic temperature, thereby improving the quality of sleep	Radhakrishnan et al. (2017)
*Yuan zhi*	*Polygala tenuifolia* Willd	Compound/Extract (water)	Polygala saponins	*In vitro* (Aβ-induced PC12 cells; BV2 cells) *In vivo* (D-galactose-induced aged mice, scopolamine-induced mice, chronic unpredictable mild stress-induced mice, APP/PS1 transgenic AD mice	Cognitive-improving effects	MAO ↓, AchE ↓, BDNF ↑, TrkB phosphorylation ↑, ASK1 ↓, JNK ↓, NT-3 ↑, NLRP3 inflammasome ↓, APP ↓, PS1/BACE1 interaction ↓	(Zhang et al., 2023)
					Improvement of synaptic transmission		
					Activation of MAPK cascades		
					Anti-apoptosis		
					Antioxidant		
		Extract (ethanol)	*Polygala tenuifolia* root extract	Neural stem cells in the hippocampal CA1 region	Therapeutic effects for insomnia, neurosis, dementia	Promotes the proliferation of neural stem cells	Park et al. (2008)
						Promotes the neurite outgrowth of rat neuronal precursor cells, HiB5	
		Compound	Tenuifolin	Freely moving mice	Sleep-improving effects: prolong the total sleep time by increasing the amount of NREM and REM sleep	Activation of the GABAergic system	Cao et al. (2016)
						Inhibition the noradrenergic system	

^a^ Herb names in *Pin yin* were standardized based on the 2020 Pharmacopoeia of the People’s Republic of China (https://db.ouryao.com/yd2020/accessed 4 September 2023).

^b^ Plant names are sourced from the “World Flora Online” (www.worldfloraonline.org accessed 4 September 2023).

“↑” indicates an upward revision; “↓” indicates a downward revision. Abbreviations: Aβ, amyloid β; Ach, acetylcholine; AChE, acetylcholinesterase; AD, Alzheimer’s disease; APP, amyloid precursor protein; ASK1, Apoptosis signal-regulating kinase 1; BACE1, beta-site amyloid precursor protein cleaving enzyme 1; BCL-2: B-cell lymphoma/leukemia-2; BDNF, brain-derived neurotrophic factor; bFGF, basic fibroblast growth factor; CAT, catalase; GABA, γ-Aminobutyric acid; GPX, glutathione peroxidase; GSH-PX, GSH, peroxidase; HIF-1, hypoxia-inducible factor −1; IL, interleukin; JNK, c-Jun N-terminal kinase; MAO, monoamine oxidase; MAPK, mitogen-activated protein kinase; MDA, malondialdehyde; NLRP3, NOD-like receptor protein 3; NO, nitric oxide; NOS, nitric oxide synthase; NREM, sleep, non-rapid eye movement sleep; NSC, neural stem cell; NT-3, neurotrophin-3; REM, sleep, rapid eye movement sleep; ROS, reactive oxygen species; SOD, superoxide dismutase; TNF-α, tumor necrosis factor α; TrkB, tropomysin related kinase B; VEGF, vascular endothelial growth factor.

### 4.4 Implication for future research

#### 4.4.1 Methodological design enhancement

It should be noted that most of the included studies were evaluated as having “some concerns” regarding the risk of bias. The main sources of bias were insufficient reporting on the randomization process, lack of pre-registered trial protocols or insufficient information on outcome measurements and statistical analysis plans provided in the trial registration. It is likely that most of the researchers were not familiar with the standards of reporting trials. Rigorous randomization can minimize the influence of other prognostic factors, while pre-registered trial protocols providing detailed information on the outcome measures as well as statistical analysis methods will improve the reliability of the study results. Researchers should pay more attention to these aspects in future studies to provide more high-quality evidence in this field.

#### 4.4.2 Quality control of CHM/placebo preparations

Rigorous quality control of herbal medicine ingredients is a critical factor for ensuring safety, maintaining a consistent phytochemical profile, and guaranteeing the clinical efficacy of these treatment (Govindaraghavan and Sucher, 2015). In our review, CHM are commonly formulated into capsules or granules to facilitate ease of administration. Placebos used in those trials were designed to mimic the appearance, taste and smell of the active treatment group, typically using food or food additives devoid of active constituents. However, the majority of the studies in our review lack a detailed description of the chemical analysis of the medicinal compounds, standardization and quality control measures for the CHM preparations and placebos, which could undermine the credibility of the findings due to the potential impact on result reproducibility. Moreover, two studies have utilized placebos consisting of active ingredients, such as low dosages of CHM used in the experimental group (Liu, 2014) or other CHM (Huang et al., 2013). For future research, it is imperative to ensure the quality of herbal medicines and their placebos by strictly adhering to Good Agricultural and Collection Practices (GACP), Good Plant Authentication and Identification Practices (GPAIP), Good Manufacturing Practices (GMP), and Good Laboratory Practices (GLP) throughout the production and analytical processes (Govindaraghavan and Sucher, 2015).

#### 4.4.3 Optimal treatment duration

Our subgroup analysis considering different treatment durations demonstrated a declining trend in the treatment effects of CHM as the duration was prolonged. Specifically, treatment durations of two to 4 months showed the maximum effect size, followed by the 6-month subgroup. However, studies with duration of 12 or 24 months exhibit minimum effect size. A similar trend was reported in previous review of CHM for MCI, which found that the 2-month study showed greater effect sizes in the outcome of MoCA compared to the 6-month studies (Dong et al., 2019). The researchers explained that the shorter studies tended to show larger effect sizes, possibly due to more pronounced test/retest effects and nonspecific benefits of participant in the initial stages of the trial (Lim et al., 2016; Hyde et al., 2017; Dong et al., 2019). Another potential explanation for this finding is that MCI is characterized by neuronal loss, synaptic degeneration (Mufson et al., 2012) and reduced cortical connectivity (Gonzalez-Escamilla et al., 2016). Early intervention may play a crucial role in preserving synaptic and neuronal function. Conversely, longer treatment duration may not necessarily provide additional benefits beyond a certain threshold, which could be associated with the brain’s compensatory mechanisms (Mufson et al., 2012). During the early stages of cognitive impairment, the brain is capable to deploy neural plasticity (Mufson et al., 2012) and activate alternative brain networks to compensate (Liang et al., 2011). However, as the disease progresses or treatment duration lengthens, these compensatory mechanisms may become depleted, resulting in cognitive decline and reduced treatment efficacy (Clement and Belleville, 2010). On the other hand, researcher indicated that clinical trials evaluating the effects of interventions on MCI symptomatic progression generally necessitate a minimum trial duration of 6 months, with a longer duration of 12 months considered optimal (Jelic et al., 2006). While the majority of studies included in this review had treatment durations of 6 months or more, it is important to recognize that the inclusion of studies with short-term treatment duration may impact the interpretation of the overall treatment effect. Moreover, the progression rate of MCI differs among various subtypes (Marra et al., 2011) Despite our efforts to explore the impact of the underlying etiology, most of the studies included in this review did not provide the information about the potential causes or subtypes of MCI. Therefore, more experimental and clinical research is needed to determine the optimal treatment duration of CHM for MCI.

Furthermore, this review revealed that despite the general long treatment duration, patients have a high acceptance of CHM as evidenced by relatively small drop-out numbers of participants. However, most studies were conducted in China, where patients are generally more receptive to CHM. Therefore, it is essential to conduct further international multi-center studies to investigate the acceptance of CHM for MCI in other countries.

#### 4.4.4 Data collection and outcome selection

The majority of the studies utilized the end-of-treatment MMSE scores as the primary outcome. However, MMSE scores can be influenced by various variables such as age, gender, education level, ethnicity, and language, as demonstrated by previous research (Escobar et al., 1986; Han et al., 2008; Solias et al., 2014). Consequently, obtaining comprehensive baseline data is crucial for interpreting the result (Solias et al., 2014). However, limited baseline information on these variables was provided by the included studies to allow us to conduct further analysis. For future research in this area, it is essential to collect and report more detailed baseline data such as age, gender and education levels. Moreover, recent studies have indicated that MoCA exhibits greater specificity and sensitivity than MMSE in evaluating of MCI (Ciesielska et al., 2016; Pinto et al., 2019). A cross-sectional study conducted within Chinese population reinforced this view, suggesting that MoCA provides a more effective assessment of cognitive function in MCI patients due to its capability to avoid ceiling effect and its proficiency in identifying cognitive heterogeneity (Jia et al., 2021). However, in this review, only three out of the thirteen included studies reported data regarding MoCA. This limited representation may stem from the MoCA’s later introduction compared to the well-established MMSE (Folstein et al., 1975; Nasreddine et al., 2005), subsequently resulting in its lower popularity and a general unfamiliarity among researchers regarding its use in clinical trials in China. In light with these findings, we recommend that future research on MCI either prioritize MoCA as the primary outcome measure or employ it alongside MMSE.

#### 4.4.5 Safety assessment and adverse events reporting

Most of the studies included in our review have provided details on safety assessment measures, including AE recording, laboratory testing, and physical examination. Nonetheless, several studies merely reported the absence of AEs during the treatment period without prescribing the methods used to monitor potential harmful outcomes (details in Supplementary Table S4). It remains uncertain whether systematic assessment of AEs was conducted across all participants through standardized clinical examinations, questionnaires, or medical instruments in those studies. Given that inadequate design may result in underestimating AEs, subsequently influencing clinical decision-making, it is crucial for future studies to pre-plan safety assessments and adhere to established standards such as CONSORT Harms and CONSORT Extension for CHM Formulas (Cheng et al., 2017; Junqueira et al., 2023). This involves considering details such as selecting outcome measures specific to safety assessments, reporting comprehensive details of all AEs (including occurrence timing, frequency, and severity), and providing interpretation regarding potential underlying causes (Cheng et al., 2017).

### 4.5 Limitations

Although efforts were made to achieve an impartial conclusion, the present study has inherent limitations. First, the presence of substantial heterogeneity among the included studies is one limitation of our meta-analysis. One potential source of heterogeneity could be the variations in participant characteristics, such as age, gender, education level or the underlying etiology of MCI. Unfortunately, due to the limited availability of individual patient data in the included studies, we were unable to perform further analysis based on these factors. Moreover, differences in the treatment protocols used in the individual trials, such as duration or different CHM ingredients, might have contributed to the observed heterogeneity.

Second, although we conducted comprehensive research to collect as many studies as possible, the number of eligible randomized placebo-controlled trials on CHM for MCI is limited. Additionally, the sample sizes for most included studies were small, and all of the studies were conducted in Asia. Therefore, it remains unclear whether the findings can be generalized to other ethnic populations. More large-scale and multi-cantered trials with detailed patient-level data are still needed to provide more robust evidence in the field of CHM for MCI.

## 5 Conclusion

In conclusion, our study suggests that CHM may serve as an effective intervention for improving cognitive function in patients with MCI, supported by low to moderate-certainty evidence. Although current data suggests CHM is generally safe, caution is advised due to the lack of AE reporting or detailed information in some instances. Notably, significant heterogeneity observed among the included studies highlights the variability, likely stemming from different CHM interventions and individual patient characteristics. In clinical practice, it is important to inform patients about the current evidence regarding CHM in MCI treatment, addressing potential benefits while considering individual patient profiles for tailored treatment plans. Further research is necessary to strengthen the evidence supporting CHM’s role in managing MCI. Studies with rigorous designs and reporting, as well as high-quality control on CHM preparations are essential.

## Data Availability

The original contributions presented in the study are included in the article/Supplementary Material, further inquiries can be directed to the corresponding authors.
