# Supplementary material for: The efficacy and safety of Chinese herbal medicine for mild cognitive impairment: a systematic review and meta-analysis of randomized placebo-controlled trials
Source: Front Pharmacol. 2024 Feb 15;15:1341074. doi: 10.3389/fphar.2024.1341074 (PMC10902497; doi:10.3389/fphar.2024.1341074)
Supplement: Supplementary file 1 [file Table1.docx]

Supplementary Material

# Supplementary Text

Supplementary Text S1. Search strategy for nine databases.

# Supplementary Tables

Supplementary Table S1. PRISMA 2020 Checklist.

Supplementary Table S2. Quality control and ingredients of CHM/placebo preparations of included studies.

Supplementary Table S3. Frequently used herbs in included studies.

Supplementary Table S4. Summary of safety assessment and adverse events of included studies.

Supplementary Table S5. GRADE assessment for MMSE and MoCA.

# Supplementary figures

Supplementary Figure S1. Risk of bias in included studies.

Supplementary Figure S2. Sensitivity analysis in terms of MMSE (one-by-one exclusion method).

Supplementary Figure S3. Forest plot for the outcome of AEs at the end of treatment.

# Supplementary Text S1. Search strategy for nine databases.

| **Databases** | **Search strategy** |
| --- | --- |
| PubMed | **#1** (Cognitive Dysfunction[MeSH Terms]) OR (Cognitive Dysfunctions[All Fields]) OR (Dysfunction, Cognitive[All Fields]) OR (Cognitive Impairments[All Fields]) OR (Cognitive Impairment[All Fields]) OR (Mild Cognitive Impairment[All Fields]) OR (Mild Cognitive Impairments[All Fields]) OR (Mild Neurocognitive Disorder[All Fields]) OR (Mild Neurocognitive Disorders[All Fields]) OR (Neurocognitive Disorder, Mild[All Fields]) OR (Neurocognitive Disorders, Mild[All Fields]) OR (Cognitive Decline[All Fields]) OR (Cognitive Declines[All Fields]) OR (Mental Deterioration[All Fields]) OR (Mental Deteriorations[All Fields]) OR (mild cognitive disorder[All Fields]) OR (mild neurocognitive impairment[All Fields]) OR (mild neurocognitive dysfunction[All Fields]) OR (minor cognitive disorder[All Fields]) OR (minor neurocognitive impairment[All Fields]) OR (minor neurocognitive decline[All Fields]) OR (minor neurocognitive disorder[All Fields])  **#2** (Medicine, Chinese Traditional[All Fields]) OR (Drugs, Chinese Herbal[All Fields]) OR (Herbal Medicine[All Fields]) OR (Medicine, Traditional[All Fields]) OR (Medicine, Kampo[All Fields]) OR (Plant Preparations[All Fields]) OR (Plants, Medicinal[All Fields]) OR (Plant Extracts[All Fields]) OR (Ethnopharmacology[All Fields]) OR (Ethnobotany[All Fields]) OR (Phytotherapy[All Fields]) OR (Materia Medica[All Fields]) OR (TCM[All Fields]) OR (Chinese AND (agent OR drug OR extract OR formula OR herb* medici* OR preparation OR prescription OR plant OR remedy OR therapy)) OR ((herb* OR botanical OR phyto) AND (agent OR drug OR extract OR materia* OR medici* OR mixture OR preparation OR product OR prescription OR plant OR remedy OR substance OR therapy)) OR (herb or herbs or herbal)  **#3** (Randomized Controlled Trial[Publication Type]) OR (randomized[Title/Abstract] AND controlled[Title/Abstract] AND trial[Title/Abstract]) OR (Controlled Clinical Trial[Publication Type]) OR (Clinical Trial[Publication Type]) OR (Clinical Trials as Topic[MeSH Terms]) OR ((clinical[Title/Abstract] AND trial[Title/Abstract]) ) OR (Drug Therapy[MeSH Terms]) OR (Therapeutic use[MeSH Subheading]) OR (Random Allocation[MeSH Terms]) OR (Random*[Title/Abstract]) OR (Placebos[MeSH Terms]) OR (Placebo[Title/Abstract])  **#4** #1 AND #2 AND #3  **#5** Animals [MeSH Terms] NOT (Humans[MeSH Terms] AND Animals[MeSH Terms])  **#6** #4 NOT #5 |
| Embase | **#1** 'mild cognitive impairment'/exp OR 'mild neurocognitive disorder'/exp OR 'mild cognitive defect'/exp OR (mild:ti,ab,kw OR minor:ti,ab,kw) AND (cognitive:ti,ab,kw OR neurocognitive:ti,ab,kw) AND (impair*:ti,ab,kw OR disorde*:ti,ab,kw OR dysfuncti*:ti,ab,kw OR declin*:ti,ab,kw OR defic*:ti,ab,kw OR defec*:ti,ab,kw) OR nmci:ti,ab,kw OR amci:ti,ab,kw OR mmci:ti,ab,kw OR cognitive deficit:ti,ab,kw OR cognitive decline:ti,ab,kw OR mental deterioration:ti,ab,kw  **#2** 'traditional medicine'/exp OR 'Chinese herb'/exp OR 'Chinese medicine'/exp OR 'Chinese drug'/exp OR 'Chinese medicinal formula'/exp OR 'herbal medicine'/exp OR 'herbaceous agent'/exp OR 'medicinal plant'/exp OR 'plant medicinal product'/exp OR 'plant extract'/exp OR 'ethnopharmacology'/exp OR 'ethnobotany'/exp OR 'phytotherapy'/exp OR 'materia medica'/exp OR 'Kampo medicine'/exp OR '(Chinese AND (agent OR drug OR extract OR formula OR herb* medici* OR preparation OR prescription OR plant OR remedy OR therapy)):ti,ab,kw OR ((herb* OR botanical OR phyto) AND (agent OR drug OR extract OR materia* OR medici* OR mixture OR preparation OR product OR prescription OR plant OR remedy OR substance OR therapy)):ti,ab,kw OR (herb or herbs or herbal):ti,ab,kw  **#3** 'randomized controlled trial'/exp OR 'random*' OR placebo* OR ((singl* or doubl* or treb* or trip*) AND (blind* or mask* or dummy))  **#4** #1 AND #2 AND #3  **#5** [animals]/lim NOT ([humans]/lim AND [animals]/lim)  **#6** #4 NOT #5 |
| CENTRAL | **#1** MeSH descriptor: [Cognitive Dysfunction] explode all trees OR MeSH descriptor: [Neurocognitive Disorders] explode all trees OR MeSH descriptor: [Cognition Disorders] explode all trees OR (Mild Cognitive Impairment):ti,ab,kw OR (Mild Cognitive Disorder):ti,ab,kw OR (Mild Neurocognitive Impairment):ti,ab,kw OR (Mild Neurocognitive Disorder):ti,ab,kw OR (Mild Neurocognitive Dysfunction):ti,ab,kw OR (Minor Neurocognitive Impairment):ti,ab,kw OR (Minor Cognitive Disorder):ti,ab,kw OR (Minor Neurocognitive Decline):ti,ab,kw OR (Minor Neurocognitive Disorder):ti,ab,kw OR (Cognitive Decline):ti,ab,kw OR (Cognitive deficit):ti,ab,kw OR (Cognitive defect):ti,ab,kw OR (Mental Deterioration):ti,ab,kw  **#2** Medicine, Chinese Traditional OR Drugs, Chinese Herbal OR Herbal Medicine OR Medicine, Traditional OR Medicine, Kampo OR Plant Preparations OR Plants, Medicinal OR Plant Extracts OR Ethnopharmacology OR Ethnobotany OR Phytotherapy OR Materia Medica OR (TCM):ti,ab,kw OR (Chinese AND (agent OR drug OR extract OR formula OR herb* medici* OR preparation OR prescription OR plant OR remedy OR therapy)):ti,ab,kw OR ((herb* OR botanical OR phyto) AND (agent OR drug OR extract OR materia* OR medici* OR mixture OR preparation OR product OR prescription OR plant OR remedy OR substance OR therapy)):ti,ab,kw OR (herb or herbs or herbal):ti,ab,kw  **#3** (Randomized Controlled Trial):pt OR (Random*):ti,ab,kw OR (placebo*):ti,ab,kw OR ((singl* or doubl* or treb* or trip*) AND (blind* or mask* or dummy)):ti,ab,kw  **#4** #1 AND #2 AND #3 [Filter:Trials] |
| CINAHL | **#1** Cognitive Dysfunction OR Cognitive Impairment OR Cognitive Decline OR Cognitive deficit OR Cognitive defect OR Mental Deterioration OR Mild Cognitive Impairment OR Mild Cognitive Disorder OR Mild Neurocognitive Impairment OR Mild Neurocognitive Disorder OR Mild Neurocognitive Dysfunction OR Minor Cognitive Disorder OR Minor Neurocognitive Impairment OR Minor Neurocognitive Decline OR Minor Neurocognitive Disorder  **#2** Medicine, Chinese Traditional OR Drugs, Chinese Herbal OR Herbal Medicine OR Medicine, Traditional OR Medicine, Kampo OR Plant Preparations OR Plants, Medicinal OR Plant Extracts OR Ethnopharmacology OR Ethnobotany OR Phytotherapy OR Materia Medica OR TCM OR (Chinese AND (agent OR drug OR extract OR formula OR herb* medici* OR preparation OR prescription OR plant OR remedy OR therapy)) OR ((herb* OR botanical OR phyto) AND (agent OR drug OR extract OR materia* OR medici* OR mixture OR preparation OR product OR prescription OR plant OR remedy OR substance OR therapy)) OR (herb OR herbs OR herbal)  **#3** Limiters: Randomized Controlled Trials  **#4** #1 AND #2 AND #3 |
| AMED | **#1** Cognitive Dysfunction OR Cognitive Impairment OR Cognitive Decline OR Cognitive deficit OR Cognitive defect OR Mental Deterioration OR Mild Cognitive Impairment OR Mild Cognitive Disorder OR Mild Neurocognitive Impairment OR Mild Neurocognitive Disorder OR Mild Neurocognitive Dysfunction OR Minor Cognitive Disorder OR Minor Neurocognitive Impairment OR Minor Neurocognitive Decline OR Minor Neurocognitive Disorder  **#2** Medicine, Chinese Traditional OR Drugs, Chinese Herbal OR Herbal Medicine OR Medicine, Traditional OR Medicine, Kampo OR Plant Preparations OR Plants, Medicinal OR Plant Extracts OR Ethnopharmacology OR Ethnobotany OR Phytotherapy OR Materia Medica OR TCM OR (Chinese AND (agent OR drug OR extract OR formula OR herb* medici* OR preparation OR prescription OR plant OR remedy OR therapy)) OR ((herb* OR botanical OR phyto) AND (agent OR drug OR extract OR materia* OR medici* OR mixture OR preparation OR product OR prescription OR plant OR remedy OR substance OR therapy)) OR (herb OR herbs OR herbal)  **#3** Randomized Controlled Trial OR Random* OR placebo* OR (singl* or doubl* or treb* or trip*) AND (blind* or mask* or dummy)  **#4** #1 AND #2 AND #3 |
| CBM | **#1**认知功能障碍 OR 轻度认知障碍 OR 轻度神经认知障碍 OR 认知减退 OR 认知损害  **#2** 中医 OR 中西医 OR 中医疗法 OR 辨病论治 OR 辨证 OR 辨证论治 OR 辨症施治 OR 汉方 OR 祖国医学 OR 传统医学 OR 传统治疗 OR 替代医学 OR 替代治疗 OR 中国传统医学 OR 民族医药 OR 草药 OR 中草药 OR 中药 OR 中药疗法 OR 中西药 OR 传统医药 OR 中成药 OR 植物药 OR 中医治法  **#3** #1 AND #2 [Filter: 随机对照试验] |
| CNKI  Wanfang  CQVIP | **#1**认知功能障碍 OR 轻度认知障碍 OR 轻度神经认知障碍 OR 认知减退 OR 认知损害  **#2** 中医 OR 中西医 OR 中医疗法 OR 辨病论治 OR 辨证 OR 辨证论治 OR 辨症施治 OR 汉方 OR 祖国医学 OR 传统医学 OR 传统治疗 OR 替代医学 OR 替代治疗 OR 中国传统医学 OR 民族医药 OR 草药 OR 中草药 OR 中药 OR 中药疗法 OR 中西药 OR 传统医药 OR 中成药 OR 植物药 OR 中医治法  **#3** 随机  **#4** #1 AND #2 AND #3 |

Abbreviations: AMED, Allied and Complementary Medicine Database; CBM, China Biomedical Literature; CENTRAL, Cochrane Central Register of Controlled Trials; CINAHL, Cumulative Index of Nursing and Allied Health Literature; CNKI, China National Knowledge Infrastructure Database; CQVIP, Chongqing VIP Database; Embase, Excerpta Medica Database.

# Supplementary Table S1. PRISMA 2020 Checklist.

| **Section and Topic** | **Item #** | **Checklist item** | **Location where item is reported (Page^a^)** |
| --- | --- | --- | --- |
| **TITLE** | | |  |
| Title | 1 | Identify the report as a systematic review. | 1 |
| **ABSTRACT** | | |  |
| Abstract | 2 | See the PRISMA 2020 for Abstracts checklist. | 1 |
| **INTRODUCTION** | | |  |
| Rationale | 3 | Describe the rationale for the review in the context of existing knowledge. | 1 |
| Objectives | 4 | Provide an explicit statement of the objective(s) or question(s) the review addresses. | 1 |
| **METHODS** | | |  |
| Eligibility criteria | 5 | Specify the inclusion and exclusion criteria for the review and how studies were grouped for the syntheses. | 2–3 |
| Information sources | 6 | Specify all databases, registers, websites, organisations, reference lists and other sources searched or consulted to identify studies. Specify the date when each source was last searched or consulted. | 3 |
| Search strategy | 7 | Present the full search strategies for all databases, registers and websites, including any filters and limits used. | 3, Text S1 |
| Selection process | 8 | Specify the methods used to decide whether a study met the inclusion criteria of the review, including how many reviewers screened each record and each report retrieved, whether they worked independently, and if applicable, details of automation tools used in the process. | 3 |
| Data collection process | 9 | Specify the methods used to collect data from reports, including how many reviewers collected data from each report, whether they worked independently, any processes for obtaining or confirming data from study investigators, and if applicable, details of automation tools used in the process. | 3 |
| Data items | 10a | List and define all outcomes for which data were sought. Specify whether all results that were compatible with each outcome domain in each study were sought (e.g. for all measures, time points, analyses), and if not, the methods used to decide which results to collect. | 3 |
|  | 10b | List and define all other variables for which data were sought (e.g. participant and intervention characteristics, funding sources). Describe any assumptions made about any missing or unclear information. | 3 |
| Study risk of bias assessment | 11 | Specify the methods used to assess risk of bias in the included studies, including details of the tool(s) used, how many reviewers assessed each study and whether they worked independently, and if applicable, details of automation tools used in the process. | 3 |
| Effect measures | 12 | Specify for each outcome the effect measure(s) (e.g. risk ratio, mean difference) used in the synthesis or presentation of results. | 3 |
| Synthesis methods | 13a | Describe the processes used to decide which studies were eligible for each synthesis (e.g. tabulating the study intervention characteristics and comparing against the planned groups for each synthesis (item #5)). | 3 |
|  | 13b | Describe any methods required to prepare the data for presentation or synthesis, such as handling of missing summary statistics, or data conversions. | 3 |
|  | 13c | Describe any methods used to tabulate or visually display results of individual studies and syntheses. | 3 |
|  | 13d | Describe any methods used to synthesize results and provide a rationale for the choice(s). If meta-analysis was performed, describe the model(s), method(s) to identify the presence and extent of statistical heterogeneity, and software package(s) used. | 3 |
|  | 13e | Describe any methods used to explore possible causes of heterogeneity among study results (e.g. subgroup analysis, meta-regression). | 3 |
|  | 13f | Describe any sensitivity analyses conducted to assess robustness of the synthesized results. | 3 |
| Reporting bias assessment | 14 | Describe any methods used to assess risk of bias due to missing results in a synthesis (arising from reporting biases). | 3 |
| Certainty assessment | 15 | Describe any methods used to assess certainty (or confidence) in the body of evidence for an outcome. | 3 |
| **RESULTS** | | |  |
| Study selection | 16a | Describe the results of the search and selection process, from the number of records identified in the search to the number of studies included in the review, ideally using a flow diagram. | 3, Figure 1 |
|  | 16b | Cite studies that might appear to meet the inclusion criteria, but which were excluded, and explain why they were excluded. | 3, Figure 1 |
| Study characteristics | 17 | Cite each included study and present its characteristics. | 3–6, Tables 1–2, Tables S2–S3 |
| Risk of bias in studies | 18 | Present assessments of risk of bias for each included study. | 6–8, Figure S1 |
| Results of individual studies | 19 | For all outcomes, present, for each study: (a) summary statistics for each group (where appropriate) and (b) an effect estimate and its precision (e.g. confidence/credible interval), ideally using structured tables or plots. | Figure 2, Figure 6 |
| Results of syntheses | 20a | For each synthesis, briefly summarise the characteristics and risk of bias among contributing studies. | 8–11, Figure 2, Figure 3, Figure 4, Figure 6 |
|  | 20b | Present results of all statistical syntheses conducted. If meta-analysis was done, present for each the summary estimate and its precision (e.g. confidence/credible interval) and measures of statistical heterogeneity. If comparing groups, describe the direction of the effect. | 8–11, Figure 2, Figure 3, Figure 4, Figure 6 |
|  | 20c | Present results of all investigations of possible causes of heterogeneity among study results. | 8–10, Figure 3, Figure 4, Table 3 |
|  | 20d | Present results of all sensitivity analyses conducted to assess the robustness of the synthesized results. | 10, Table 4, Figure S2 |
| Reporting biases | 21 | Present assessments of risk of bias due to missing results (arising from reporting biases) for each synthesis assessed. | 10–11, Figure5 |
| Certainty of evidence | 22 | Present assessments of certainty (or confidence) in the body of evidence for each outcome assessed. | 11–12, Table S5 |
| **DISCUSSION** | | |  |
| Discussion | 23a | Provide a general interpretation of the results in the context of other evidence. | 12 |
|  | 23b | Discuss any limitations of the evidence included in the review. | 12, 15–16 |
|  | 23c | Discuss any limitations of the review processes used. | 16 |
|  | 23d | Discuss implications of the results for practice, policy, and future research. | 12, 15–16 |
| **OTHER INFORMATION** | | |  |
| Registration and protocol | 24a | Provide registration information for the review, including register name and registration number, or state that the review was not registered. | 2 |
|  | 24b | Indicate where the review protocol can be accessed, or state that a protocol was not prepared. | 2 |
|  | 24c | Describe and explain any amendments to information provided at registration or in the protocol. | NA |
| Support | 25 | Describe sources of financial or non-financial support for the review, and the role of the funders or sponsors in the review. | 16 |
| Competing interests | 26 | Declare any competing interests of review authors. | 17 |
| Availability of data, code and other materials | 27 | Report which of the following are publicly available and where they can be found: template data collection forms; data extracted from included studies; data used for all analyses; analytic code; any other materials used in the review. | 16 |

Abbreviation: NA, not applicable. ^a^Page refers to the page number in the original submitted materials.

# Supplementary Table S2. Quality control and ingredients of CHM/placebo preparations of included studies.

| **Study** | **CHM preparations;**  **Pharmaceutical producer** | **CHM ingredients (*Pin yin* names^a^ & plant names^b^)** | **Placebo ingredients; Pharmaceutical producer of placebo** | **Quality control measure** |
| --- | --- | --- | --- | --- |
| Zhou 2007 | *Shen yin* oral solution (参银口服液); Institute of Shanghai Traditional Chinese Geriatrics, China (10 mL/支，含生药量19.5g，由上海市中医老年医学研究所提供) | ***Pin yin* names:** *Dang shen, Yin xing ye, Long yan rou, Da zao,* etc.  **Plant names:** *Salvia miltiorrhiza* Bunge., Ginkgo biloba L., *Dimocarpus longan* Lour., *Ziziphus jujuba* Mill. | Jujube flavour, distilled water; Institute of Shanghai Traditional Chinese Geriatrics, China (包装、色泽、剂量与中药组相同，10 mL/支，由上海市中医老年医学研究所提供) | NS |
| Wu 2010 | *Tian tai* No.1 capsule (天泰1号胶囊); Shenzhen Traditional Chinese Medicine Co., Ltd (每粒胶囊0.5g，相当于生药6.3g，由深圳市中药总厂生产，批号：030601) | ***Pin yin* names:** *Tian ma, Ren shen,* etc.  **Plant names:** *Gastrodia elata* Blume, *Panax ginseng* C.A.Mey. | Starch and excipient; Shenzhen Traditional Chinese Medicine Co., Ltd (与治疗组药物的形状、包装、颜色及剂量完全相同的仿照品，由深圳市中药总厂生产，批号：030602) | NS |
| Dai 2011 | *Yi zhi jian nao* granule (益智健脑颗粒); Preparation Room of Zhejiang Provincial Hospital of Traditional Medicine, China (浙江省中医院制剂室制备提供，批号：20060807) | ***Pin yin* names:** *Gou qi zi, Huang jing, Yi zhi ren, Shu di, Huang qi, Yu jin, Tao ren, Chang pu, Yuan zhi*  **Plant names:** *Lycium barbarum* L., *Polygonatum kingianum* Collett & Hemsl., *Alpinia oxyphylla* Miq., *Rehmannia glutinosa* (Gaertn.) DC., *Astragalus mongholicus* Bunge, *Curcuma wenyujin* Y.H.Chen & C.Ling, *Prunus persica* (L.) Batsch, *Acorus calamus* var. *angustatus* Besser, *Polygala tenuifolia* Willd. | No information; Preparation Room of Zhejiang Provincial Hospital of Traditional Medicine, China (同治疗组药物的形状、包装、颜色及剂量完全相同的不含药物仿照品，由浙江省中医院制剂室制备提供，批号: 20060808) | NS |
| Huang 2013 | *Nao kang* II decoction (脑康Ⅱ号汤剂); NS | ***Pin yin* names:** *Zhi shou wu, Shu di huang, San qi, Chang pu, Yuan zhi*  **Plant names:** *Polygonum multiflorum* Thunb*.*, *Rehmannia glutinosa* (Gaertn.) DC., *Panax notoginseng* (Burkill) F.H.Chen, *Acorus calamus* var. *angustatus* Besser, *Polygala tenuifolia* Willd. | Medicated leaven (*Shen qu*) (stir-fried), bittering agent, food colouring; NS | NS |
| Su 2013 | *Jian pi tian jing* formula (granule) (健脾填精方颗粒剂); Jiangyin Tianjiang Pharmaceutical Co., Ltd. (江苏江阴制药有限公司生产，批号：1105346) | ***Pin yin* names:** *Ren shen, Ba ji tian,* *Bai zhu, Huang lian, Tian ma,* etc.  **Plant names:** *Panax ginseng* C.A.Mey., *Morinda officinalis* F.C.How, *Atractylodes macrocephala* Koidz., *Coptis chinensis* Franch., *Gastrodia elata* Blume | No information; Jiangyin Tianjiang Pharmaceutical Co., Ltd. (江苏江阴制药有限公司生产) | NS |
| Liu 2014 | *Xiao xu ming* decoction (小续命方汤药); NS | ***Pin yin* names:** *Ma huang, Gui zhi, Fang feng, Fang ji, Xing ren, Huang qin, Ren shen, Fu zi, Chuan xiong, Bai shao, Sheng jiang, Gan cao*  **Plant names:** *Ephedra sinica* Stapf, Cinnamomum cassia (L.) J.Presl, *Saposhnikovia divaricata* (Turcz.) Schischk., *Stephania tetrandra* S.Moore, *Prunus armeniaca* L., *Scutellaria baicalensis* Georgi, *Panax ginseng* C.A.Mey., *Aconitum carmichaelii* Debeaux, *Ligusticum striatum* DC., *Paeonia lactiflora* Pall., *Zingiber officinale* Roscoe, *Glycyrrhiza uralensis* Fisch. | Low dosage of the CHM decoction used in the CHM group (10%); NS | NS |
| Shi 2015 | *Yi zhi jian* granule (益智煎免煎颗粒); NS | ***Pin yin* names:** *Shan zhu yu, Huang jing, Zhi he* *shou wu,* *Shi chang pu, Hong shen,* *Chuan xiong*  **Plant names:** *Cornus officinalis* Siebold & Zucc., *Polygonatum kingianum* Collett & Hemsl., *Polygonum multiflorum* Thunb*.*, *Acorus calamus* var. *angustatus* Besser, *Panax ginseng* C.A.Mey., *Ligusticum striatum* DC. | Starch (益智煎模拟免煎颗粒); NS | NS |
| Wu 2016 | *Bu shen jian pi hua tan* pill (补肾健脾化痰丸); Beijing Kangrentang Pharmaceutical Co., Ltd. (由北京康仁堂药业有限公司生产) | No information | No information; Beijing Kangrentang Pharmaceutical Co., Ltd. (由北京康仁堂药业有限公司生) | NS |
| Zhang 2016 | *Bu shen* capsule; NS (3 times a day, 4 capsules a time) | ***Pin yin* names:** *Zhi he shou wu, He ye,* *Rou cong rong, Di long, Lou lu*  **Plant names:** *Polygonum multiflorum* Thunb, *Nelumbo nucifera* Gaertn*., Cistanche deserticola* Ma, *Pheretima aspergillum*(E.Perrier), *Rhaponticum uniflorum* DC. | No information; NS; (3 times a day, 4 capsules a time) | NS |
| Park 2019 | Panax ginseng powder; Panax ginseng (4-year-old Panax ginseng) was supplied by the Chungbuk Ginseng Cooperative Association (the ginseng contains total 53 mg/g ginsenoside) powdered and encapsulated (300 mg/1 capsule) by HANIL PFC. Co., Ltd. | ***Pin yin* name:** *Ren shen*  **Plant name:** *Panax ginseng* C.A.Mey. | Starch; NS | NS |
| Lu 2020 | *Deng zhan sheng mai* capsule; Yunnan Biovalley Pharmaceutical Co., Ltd. (Yunnan, China) | ***Pin yin* names:** *Deng zhan xi xin, Ren shen, Wu wei zi, Mai men dong*  **Plant names:** *Erigeron breviscapus* (Vaniot) Hand.-Mazz., *Panax ginseng* C.A.Mey., *Schisandra chinensis* (Turcz.) Baill., *Ophiopogon japonicus* (Thunb.) Ker Gawl. | Corn starch, caramel pigment, food colouring (milk chocolate brown & lemon yellow), bittering agent; Yunnan Biovalley Pharmaceutical Co., Ltd | NS |
| Chen 2021 | MLC901 capsule (each 0.4 g of capsule contains extracts of 9 herbal components); Moleac Pte Ltd. | ***Pin yin* names:** *Huang qi, Dan shen, Chi shao, Chuan xiong, Dang gui, Hong hua, Tao ren, Yuan zhi, Shi chang pu*  **Plant names:** *Astragalus mongholicus* Bunge, *Salvia miltiorrhiza* Bunge, *Paeonia lactiflora* Pall., *Ligusticum striatum* DC., *Angelica sinensis* (Oliv.) Diels, *Carthamus tinctorius* L., *Prunus persica* (L.) Batsch, *Polygala tenuifolia* Willd., *Acorus calamus* var. *angustatus* Besser | Barley, dried ripe fruit, noodle fish and citric acid; Moleac Pte Ltd. | NS |
| Shin 2021 | *Kami-guibi-tang* *granule*; Kyoung Bang Pharmaceutical Co., Ltd. (Incheon, Korea) | ***Pin yin* names:** *Ren shen, Bai zhu, Fu ling,* *Huang qi, Long yan rou, Suan zao ren, Chai hu, Bai zhi,* *Yuan zhi, Zhi zi, Mu dan pi, Da zao, Mu xiang,* *Gan cao,* *Sheng jiang*  **Plant names:** *Panax ginseng* C.A.Mey., *Atractylodes macrocephala* Koidz., *Wolfiporia cocos* (Schw.) Ryv. & Cilbn., *Astragalus mongholicus* Bunge, *Dimocarpus longan* Lour., *Ziziphus jujuba* var. *spinosa* (Bunge) Hu ex H.F.Chow., *Bupleurum chinense* DC., *Angelica dahurica* (Hoffm.) Benth. & Hook.f. ex Franch. & Sav., *Polygala tenuifolia* Willd., *Gardenia jasminoides* J.Ellis, *Paeonia suffruticosa* Andrews, *Ziziphus jujuba* Mill., *Aucklandia costus* Falc., *Glycyrrhiza uralensis* Fisch., *Zingiber officinale* Roscoe | Corn starch, lactose, hydroxypropyl cellulose, caramel colour (food additives), tartrazine (FD&C Yellow 5), Allura Red AC (FD&C Red 40), and *Ssanghwa* flavour. Kyoung Bang Pharmaceutical Co., Ltd. (Incheon, Korea) | Using the standard method of manufacturing according to the Korean Good Manufacturing Practice guidelines. |

^a^ Herb names in *Pin yin* were standardized based on the 2020 Pharmacopoeia of the People’s Republic of China (<https://db.ouryao.com/yd2020/> accessed 4 September 2023). Herbs from the same part of a plant that were processed in different ways or were named differently over time, were identified as one same herb during frequency analysis. However, the original names of the herbs were kept in this ingredients table. ^b^ Plant names are sourced from the “World Flora Online” ([www.worldfloraonline.org](http://www.worldfloraonline.org) accessed 4 September 2023).
Abbreviations: CHM: Chinese herbal medicine; NS: not specified.

# Supplementary Table S3. Frequently used herbs in included studies.

| **Herb name in *Pin yin^a^*** | **Number of studies** | **Plant names^b^** |
| --- | --- | --- |
| *Ren shen* | 7 | *Panax ginseng* C.A.Mey. |
| *Shi chang pu* | *4* | *Acorus calamus* var. *angustatus* Besser |
| *Yuan zhi* | 4 | *Polygala tenuifolia* Willd. |
| *Chuanxiong* | 3 | *Ligusticum striatum* DC. |
| *Huang qi* | 3 | *Astragalus mongholicus* Bunge |
| *Zhi he shou wu* | 3 | *Polygonum multiflorum* Thunb. |
| *Bai zhu* | 2 | *Atractylodes macrocephala* Koidz. |
| *Da zao* | 2 | *Ziziphus jujuba* Mill. |
| *Gan cao* | 2 | 1. *Glycyrrhiza uralensis* Fisch.  2. *Glycyrrhiza inflata* Batalin  3. *Glycyrrhiza glabra* L*.* |
| *Huang jin* | 2 | 1. *Polygonatum kingianum* Collett & Hemsl.  2. *Polygonatum sibiricum* F.Delaroche  3. *Polygonatum cyrtonema* Hua |
| *Long yan rou* | 2 | *Dimocarpus longan* Lour. |
| *Sheng jiang* | 2 | *Zingiber officinale* Roscoe |
| *Shu di huang* | 2 | *Rehmannia glutinosa* (Gaertn.) DC. |
| *Tao ren* | 2 | 1. *Prunus persica* (L.) Batsch  2. *Prunus davidiana* (Carrière) N.E.Br. |
| *Tian ma* | 2 | *Gastrodia elata* Blume |

^a^ Herb names in *Pin yin* were standardized based on the 2020 Pharmacopoeia of the People’s Republic of China (<https://db.ouryao.com/yd2020/> accessed 4 September 2023). Herbs from the same part of a plant that were processed in different ways or were named different over time, were identified as one same herb during frequency analysis.
^b^ Plant names are sourced from the “World Flora Online” ([www.worldfloraonline.org](http://www.worldfloraonline.org) accessed 4 September 2023)

# Supplementary Table S4. Summary of safety assessment and adverse events of included studies.

| **Study** | **Safety assessment measures** | **Safety assessment results** | **AEs/SAEs reported in each group** | |
| --- | --- | --- | --- | --- |
|  |  |  | **CHM group** | **placebo group** |
| Zhou 2007 | 1. Physical examinations 2. Laboratory tests 3. ECG 4. AEs | No significant abnormality was found in the examinations before and after treatment in both groups. No SAEs were observed in either group. | 0 | 1 (2.7%) experienced rash and itching (symptoms disappeared without treatment). |
| Wu 2010 | 1. Physical examinations 2. Laboratory tests 3. ECG 4. AEs | No significant change was found in the examinations before and after treatment in both groups. | 0 | 0 |
| Dai 2011 | No specific safety assessment measure was mentioned in the method | No AEs were observed during the treatment duration. | 0 | 0 |
| Huang 2013 | TESS (Toxicity, laboratory tests, nervous system, cardiovascular system assessment) | There is no statistically significant difference between the two groups for each item of the TESS score. | Main AEs included dry mouth, constipation, and headache; no special treatment was given to both groups. | |
| Su 2013 | 1. Vital sings 2. Laboratory tests 3. AEs (clinical symptoms, measurement indicators, severity, treatment) | Neither group exhibited any AEs during the treatment. No abnormal changes linked to intervention were detected in the examinations conducted before and after treatment. | 0 | 0 |
| Liu, 2014 | AEs | None of the participants had SAEs. | 1 (4.17%) participant reported gastrointestinal discomfort | 0 |
| Shi 2015 | 1. Vital signs 2. Laboratory tests 3. ECG 4. AEs | No AEs were observed in either group. | 0 | 0 |
| Wu 2016 | 1. Laboratory tests 2. ECG 3. AEs | No significant change was found in the examinations before and after treatment in both groups. | 1 (1.6 %) participant experienced toothache (symptoms disappeared without treatment).  1 (1.6%) experienced constipation (symptoms disappeared without treatment). | 0 |
| Zhang 2016 | 1. Physical examinations 2. Laboratory tests 3. AEs | The incidence of AEs, results of laboratory tests, and physical examinations showed no significant differences between the two groups. | 1 (3.3%) participant experienced a decreased appetite (received no treatment, and the symptom faded away 4 days later).  7 (23.3%) participants experienced constipation. | 1 (3.3%) participant experienced mild nausea (received no treatment and the symptom faded away 4 days later)  4 (13.3%) participants experienced constipation. |
| Park 2019 | 1. Vital signs 2. Laboratory tests 3. AEs | None of the AEs were related to the CHM (ginseng) administration. These AEs were mild or moderate and transient. | 1 (2.2%) participant showed a mild increase in thyroid-stimulating hormone.  1 (2.2%) participant underwent polypectomy. | 1 (2.2%) participant showed a moderate increase in thyroid-stimulating hormone. |
| Lu 2020 | Not mentioned | Not applicable | No information | No information |
| Chen 2021 | 1. AEs 2. SAEs (SAE criteria are: death/life threatening/prolonged hospitalization, incapacity/important medical event) | There was no significant difference between the CHM and placebo group in terms of the proportion of participants experiencing AE or SAE.  A total of six AEs were possibly related to the administration of the CHM intervention: dry throat, dizziness, hematoma right thigh, vomiting, neuropathic pain and sleepiness. | 32 (56%) participants experienced AEs (including SAEs)  13 (22.8%) participants experienced SAEs | 20 (43%) participants experienced AEs (including SAEs)  6 (13.0%) participants experienced SAEs |
| Shin 2021 | 1. Vital signs 2. Laboratory tests 3. ECG 4. Brain MRI 5. AEs (The severity and relationship of the AEs to the study drug were assessed) | The frequency of AEs was not significantly different between the two groups, and there were no abnormalities in vital signs or blood tests, ECG, and brain MRI findings after the intervention. There was no link found between these AEs and the study drug. | 1 (5.9%) participant experienced temporomandibular joint pain.  1 (5.9%) participant experienced mild dyspepsia.  These symptoms appeared after taking the medication for more than 12 weeks and subsided spontaneously within 2 weeks. | 1 (6.3%) participant experienced heartburn.  1 (6.3%) experienced developed gastric cancer. |

Abbreviations: AEs, adverse events; CHM, Chinese herbal medicine; ECG, Electrocardiogram; MRI, magnetic resonance imaging; SAEs, serious adverse events; TESS, Treatment Emergent Symptom Scale.

# Supplementary Table S5. GRADE assessment for MMSE and MoCA.

| **Outcomes** | **No. RCTs** | **No. participants** | **MD [95%CI], I^2^** | **Certainty of the evidence (GRADE)** |
| --- | --- | --- | --- | --- |
| MMSE | 11 | 823 | 1.90 [1.22, 2.58], 87% | Moderate ^a^ |
| MoCA | 3 | 241 | 2.88 [1.69, 4.06], 81% | Low ^a, b^ |

Abbreviations: CI: confidence interval; I^2^: index of heterogeneity; MD, mean difference; MMSE, Mini-Mental State Examination; MoCA, Montreal Cognitive Assessment; RCT: randomized controlled trial.
^a^ Downgraded 1 level due to inconsistency.
^b^ Downgraded 1 level due to imprecision.

# Supplementary Figure S1. Risk of bias in included studies.


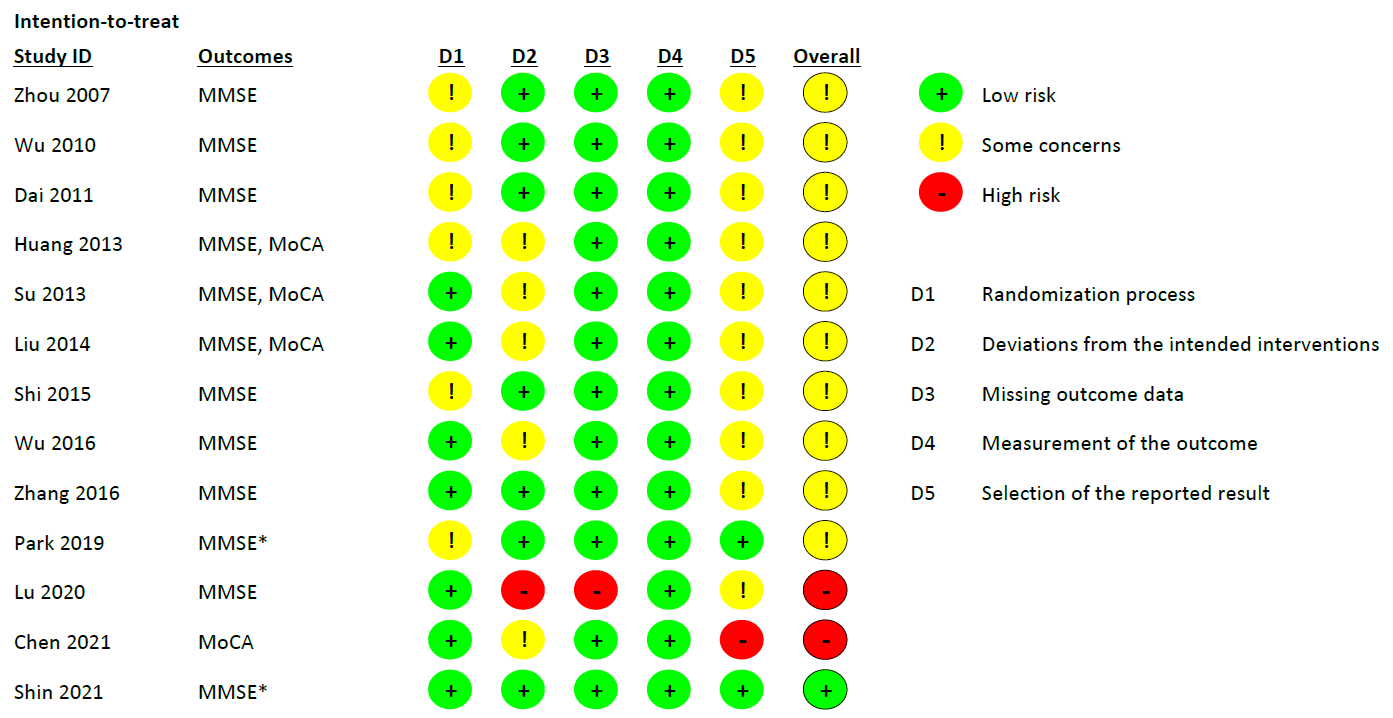


**Supplementary Figure S1.**

Risk of bias in included studies.

Abbreviations: MMSE, Mini-Mental State Examination; MMSE*: Korean version Mini-Mental State Examination; MoCA, Montreal Cognitive Assessment.

# Supplementary Figure S2. Sensitivity analysis in terms of MMSE (one-by-one exclusion method).


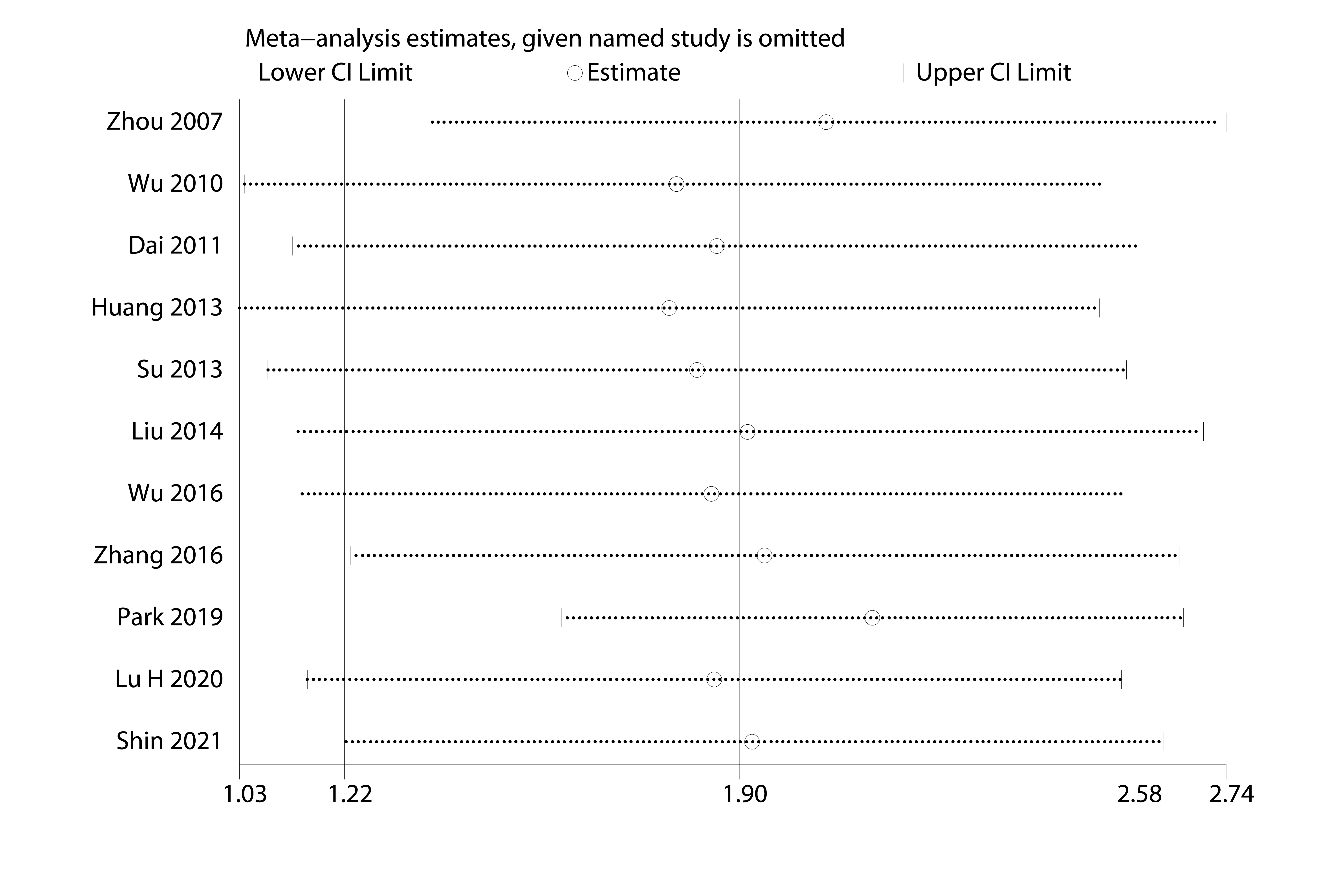


**Supplementary Figure S2.**

Sensitivity analysis in terms of MMSE (one-by-one exclusion method).

# Supplementary Figure S3. Forest plot for the outcome of AEs at the end of treatment.


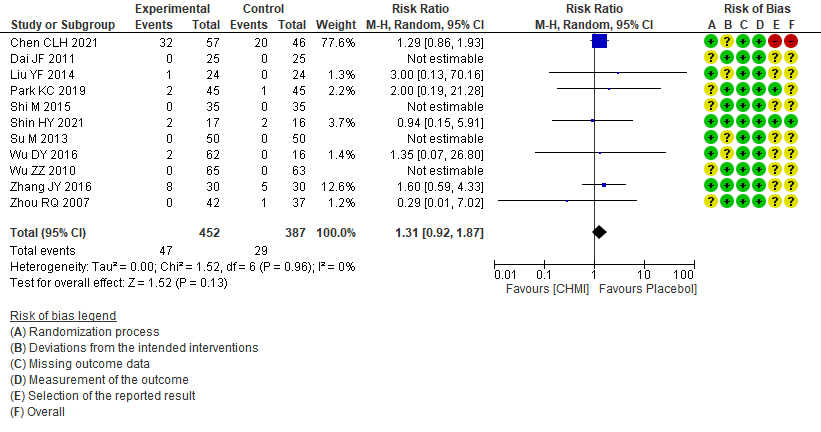


**Supplementary Figure S3.**

Forest plot for the outcome of AEs at the end of treatment.

Abbreviations: AEs, adverse events; CHM, Chinese herbal medicine.
